# Comparison of conventional and radiomics-based analysis of myocardial infarction using multimodal non-linear optical microscopy

**DOI:** 10.1038/s41598-025-10515-y

**Published:** 2025-07-18

**Authors:** Gabriel Giardina, Laszlo Papp, Arno Krause, James Marchant, Nestor Pallares-Lupon, Kanchan Kulkarni, Clemens P. Spielvogel, David Haberl, Xu Li, Wolfgang Drexler, Richard D. Walton, Angelika Unterhuber, Marco Andreana

**Affiliations:** 1 Center for Medical Physics and Biomedical Engineering, Medical University of Vienna, Vienna, Austria; 2 IHU Liryc, Univ. Bordeaux, INSERM, CRCTB, U 1045, Bordeaux, France; 3 Division of Nuclear Medicine, Department of Biomedical Imaging and Image-Guided Therapy, Medical University of Vienna, Vienna, Austria

**Keywords:** Second Harmonic Generation, Two-Photon Fluorescence, Myocardial Infarction, Scar Tissue, IBSI Radiomics, Machine Learning, Biophotonics, Scientific data, Heart failure

## Abstract

Myocardial infarction, a leading cause of mortality worldwide, leaves survivors at significant risk of recurrence caused by scar-related re-entrant ventricular tachyarrhythmias. Effective treatment with ablation therapy requires a precise guidance system. Non-linear optical microscopy techniques, such as second harmonic generation (SHG) and two-photon excited fluorescence (TPEF), are promising candidates for a high-resolution alternative to conventional electrical mapping for assessing infarcted cardiac tissue. Here, we apply SHG and TPEF with a resolution advantage over commonly used electrical mapping techniques to assess ex-vivo sheep heart infarction. Analyzing conventional and radiomic features allows for quantitative characterization of scar tissue. Our machine learning classifier achieved high accuracy, offering a promising, data-driven approach for guiding in-situ ablation therapy with increased precision. This study represents a significant step towards integrating quantitative image analysis in therapeutic interventions.

## Introduction

The World Health Organization estimates that around 7 million people die from myocardial infarction (MI) each year, making it the leading cause of death worldwide^1^. In recent years, advances in the treatment of acute MI have allowed to reduce the mortality rate down to 10% in developed countries^2^. However, around 20% of the surviving patients will experience recurrent MI within one year. Up to 50% of major coronary events occur in patients with a history of prior MI and the mortality rate of this patient population remains high^3^. Understanding the recovery process after the first acute MI is, therefore, of a high priority to address the causes of recurring MI and improve patient outcomes.

Following the occurrence of the first MI, the infarcted site undergoes a series of healing and remodeling processes in order to preserve cardiac function^4^. Necrosis of the affected cardiomyocytes occurs during the acute stage of MI and triggers an inflammatory response which takes place over the first week following the acute stage in humans and large animals. As the necrotic cardiomyocytes are resorbed, a temporary granulation tissue consisting of a variety of matrix molecules provides the necessary structural substrate to maintain cardiac function until new collagen fibers can shoulder the mechanical load. Fibrosis starts a week after the acute stage and lasts several weeks in humans and large animals. It is characterized by the recruitment, proliferation and up-regulation of cardiofibroblasts in the infarcted area. Their number increases and they produce collagen fibrils at high rates, progressively replacing the temporary granulation tissue. As this happens, and under the mechanical load caused by the contractions of the heart, the collagen fibrils arrange into stronger collagen fibers. Starting around 6 weeks after the acute stage, apoptosis of cardiofibroblasts increases and their number declines in the infarcted area causing the collagen content to stabilize. This maturation and remodeling stage can last for months in large animals and humans to develop the mature scar^5,6^. Scar tissue allows the heart to maintain its pump function^7^. However, because collagen acts as an insulator, fibrosis electrically uncouples the myocardium, resulting in conduction discontinuities which promote arrhythmia. The current literature attributes recurrent MI in part to the effect of chronic re-entrant ventricular tachyarrythmias caused by small strands of surviving myocardium through the scar^8,9^. This can nowadays be treated with ablation therapy, a surgical intervention that aims to restore normal conductive behavior in the heart by locally destroying the tissue responsible for the re-entrant arrhythmia^10^.

Ablation therapy is typically guided by electro-anatomical mapping, which relies on anatomical landmarks or surrogate representations of the arrhythmogenic substrate, such as voltage or activation maps. While recent advances have improved spatial resolution, these approaches often remain limited in their ability to capture the complex microstructural and electrophysiological heterogeneity of scarred myocardium. In particular, electrograms recorded within fibrotic tissue frequently display ambiguous signal morphologies, lack pathological specificity, and are prone to low signal-to-noise ratios, complicating precise delineation of ablation targets. The underlying tissue composition giving rise to these abnormal electrograms remains poorly defined in both research and clinical settings. The micro-structural tissue composition responsible for abnormal electrical signals lacks definition, in controlled research conditions or in clinical practice. Microscale imaging of the subsurface tissue architecture and composition would represent a paradigm-shift in curative ablation therapy guidance^11^.

Current clinical imaging techniques, including echo-cardiography, computed tomography (CT), positron emission tomography (PET), and cardiac magnetic resonance imaging (CMR), play a critical role in identifying structural substrates of arrhythmia. Among these, CMR is widely regarded as the gold standard for non-invasive scar visualization due to its sensitivity to tissue composition. However, its clinical utility remains constrained by limited spatial resolution, incompatibility with implanted cardiac devices, and insufficient sensitivity to detect micro-structural features such as narrow isthmuses of residual myocardium or discontinuous collagen deposition. More fundamentally, these imaging modalities do not offer direct visualization of the cellular or extracellular matrix organization responsible for arrhythmogenicity.

In recent years, non-linear optical microscopy (NLOM) techniques such as second harmonic generation (SHG) and two photon excited fluorescence (TPEF) showed much promise for ex-vivo cardiac tissue imaging^12,13^. SHG is a powerful tool to image endogenous collagen in tissue with high specificity without the need for staining and has emerged as one of the main optical methods to image and study the extracellular matrix^14,15^. On the other hand, TPEF targets endogenous fluorescent molecules inside the cardiomyocytes and provides complementary contrast^16^. This multimodal approach is able to image cardiac tissue using only endogenous contrast at the cellular scale and at higher speed compared to electro-anatomical mapping. This would, in principle, make it suitable for in-vivo measurements, for instance using fiber optics endoscopes, and thus guide the surgery with much better resolution compared to current techniques.

The current literature agrees that collagen amount, the morphology and the orientation of collagen fibers are the hallmarks of scar tissue resulting from MI. However, there is currently no consensus in the literature which quantitative imaging markers are the most significant and different works propose their own set of features based on different processing steps and mathematical definitions^17–21^.

The question of describing and extracting the most relevant imaging features in an objective, quantitative and reproducible way has been a major challenge in other fields of medical imaging such as CT, PET and magnetic resonance imaging (MRI). There, significant efforts have been made by the medical imaging community to establish a mathematically objective way to quantitatively describe images independently of the imaging modality used. This resulted in the proposition of the Image Biomarker Standardisation Initiative’s (IBSI) radiomic features, a consensus-based, exhaustive and robust set of mathematical definitions of all the relevant ways to quantitatively describe an image^22,23^. To date, radiomics have been extensively investigated in cancer research, primarily for multimodal PET, CT and MRI hybrid imaging analyses^24^. Radiomics have the advantage of being mathematically objective and, in contrast to more conventional imaging markers, can be extracted automatically from the images making them suitable to treat large amounts of images required by data-driven methods such as machine learning. In particular, mixed-stacked ensemble models, also called super-learners, are reportedly the most powerful method to build high-performing prediction models when relying on tabular data such as radiomics. They not only build a heterogeneous set of different machine learning models, but also perform automated data preprocessing within one integrated framework. This eliminates tedious optimization approaches and inherently addresses the issue of indirect data leakages that are results of internal test-re-test approaches until machine learning models reach an acceptable performance, and typically performed by the researcher. With this technique, no human intervention is necessary to tweak either the data or the involved machine learning hyperparameters, as the whole process is performed automatically in a controlled way where no data leakage occurs. More recently, strides have been made in the adoption of IBSI radiomics in the field of microscopy. In a previous study, our group demonstrated the application of radiomics to describe and classify multimodal NLOM images of pituitary gland and adenomas^25^. However, the main downside of radiomics is their level of abstraction which makes them difficult to interpret directly for biologists and clinicians. In this sense, more work is required to link IBSI radiomic features to more conventional image analysis methods and to the underlying biology.

In this study, we demonstrate a systematic approach to the image processing and analysis of 262 multimodal microscopy images including SHG and TPEF of an ovine heart infarction model. On each image, we analyzed 16 conventional imaging features found in the literature based on collagen amount, fiber morphology and orientation. In parallel, we extracted the full set of standardized IBSI radiomic features from the same images. By calculating the linear correlation between these two sets of features, we are able to give meaning to the more abstract radiomic features by linking them to the more straightforward conventional features and thus provide the necessary biological context. Finally, we trained a mixed-stacked ensemble machine learning classifier on the features extracted from a subset of 92 multimodal images to recognize the different histological states. This classifier showed a top-level, three-class accuracy of 95% ± 4.5% after 100-fold internal validation on the 92 training images. This model was then tested against an independent test comprised of 170 images from a different animal, demonstrating a three-class accuracy of 42% and a binary accuracy of 89%. To our knowledge, this is the first time such a quantitative approach has been employed to describe the scarring process in MI, which could pave the way to data driven methods for guiding ablation therapy in-situ.

## Results

### Imaging results

**Figure 1:**
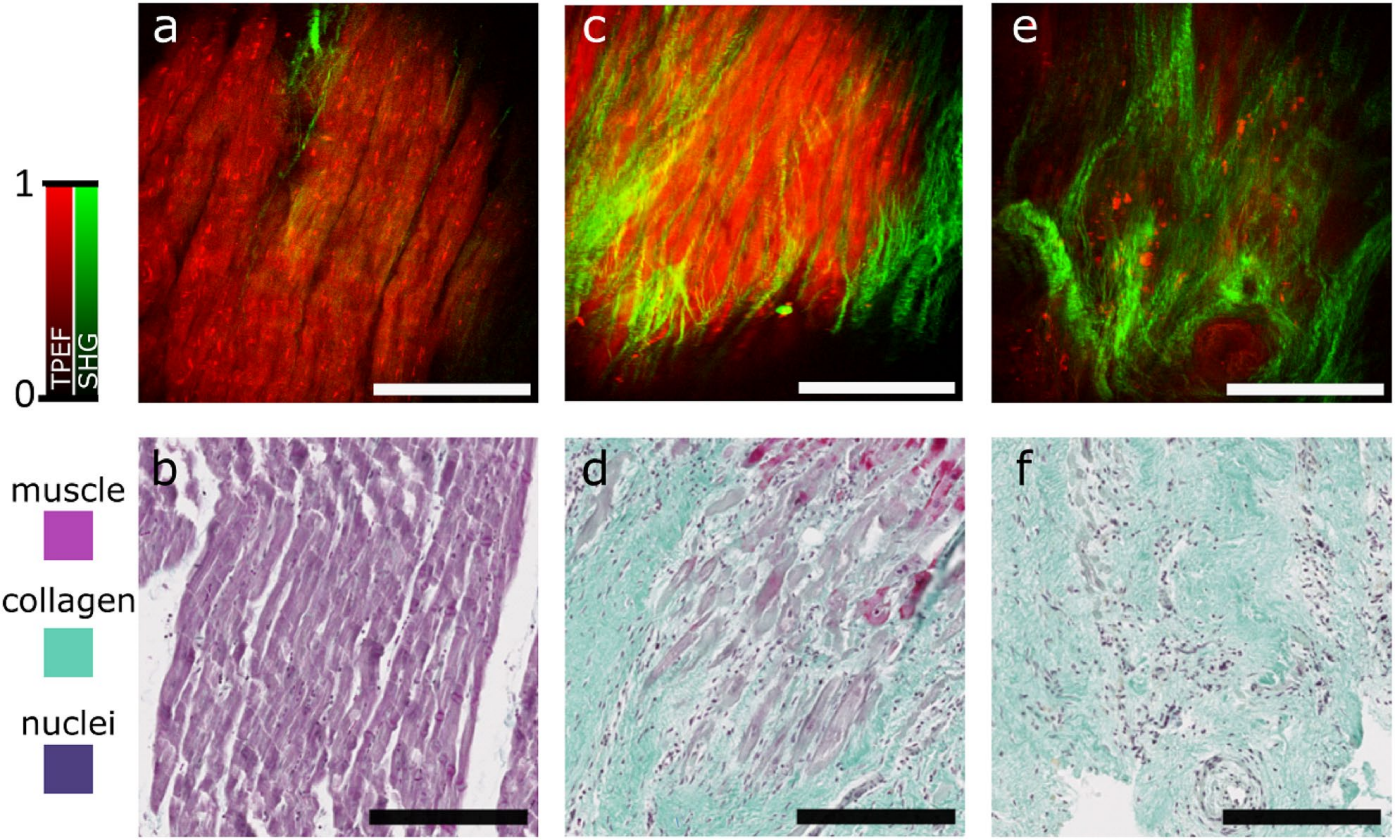
Representative NLOM images of each of the 3 classes with TPEF (in red) and SHG (in green) channels and the corresponding Masson’s trichrome stained microscopy images used for the histological assesment. (**a**,**b**) Muscle tissue, (**c**,**d**) border tissue, (**e**,**f**) fibrotic tissue. All scale bars are 200 $\upmu$m.

We performed multimodal NLOM imaging of 3 myocardium samples. Figure 1 displays representative NLOM images for each class of tissue (healthy muscle, fibrosis and border tissue) and the corresponding histology image. We imaged between 2 and 3 regions of interest (ROIs) for each sample (8 ROIs in total). At each ROI, we acquired images at 5 $\upmu$m intervals in depth starting from the surface.

For the 2 muscle ROIs we acquired 28 and 39 images respectively; for the 4 border ROIs 17, 34, 34 and 27 images and for the 2 fibrosis ROIs 30 and 53 images, for a total of 262 multimodal NLOM images. The samples were then stained using a standard Masson’s trichrome protocol and imaged with conventional light microscopy for histological assessment.

The images from healthy ROIs (Fig. 1a,b) display long, parallel muscle fibers typical for myocardium. The intensity of the TPEF channel (in red), which is sensitive to endogenous fluorescent molecules present in the cardiomyocytes, dominates the image. The image also shows a few thin and short collagen strands in the SHG channel (in green) in-between the myocardial fibers.

The images from the border ROIs (Fig. 1c,d) show the inflammatory and remodeling responses as they are taking place. In the trichrome image (Fig. 1d) the nuclei of the many immune cells and cardiofibroblasts recruited to the infarction site during the inflammatory response are visible. In Fig. 1c, a fiber-like structure is still visible in the TPEF channel but not as neatly arranged as in the healthy muscle tissue and the interstitial space is filled with collagen fibers visible in the SHG channel. Compared to the healthy tissue, the collagen fibers are more numerous, longer and thicker. They also tend to interlace with one another instead of running parallel to the myocardial fibers.

The images from the fibrotic ROIs (Fig. 1e,f) show the mature scar. The TPEF intensity is very low and the remaining myocardium is very sparse and amorphous. The image is dominated by the contribution of the SHG channel where thick, long collagen fibers take up most of the field of view. The fibers are very interlinked with one another and they form a mesh-like structure.

### Conventional imaging features

**Figure 2:**
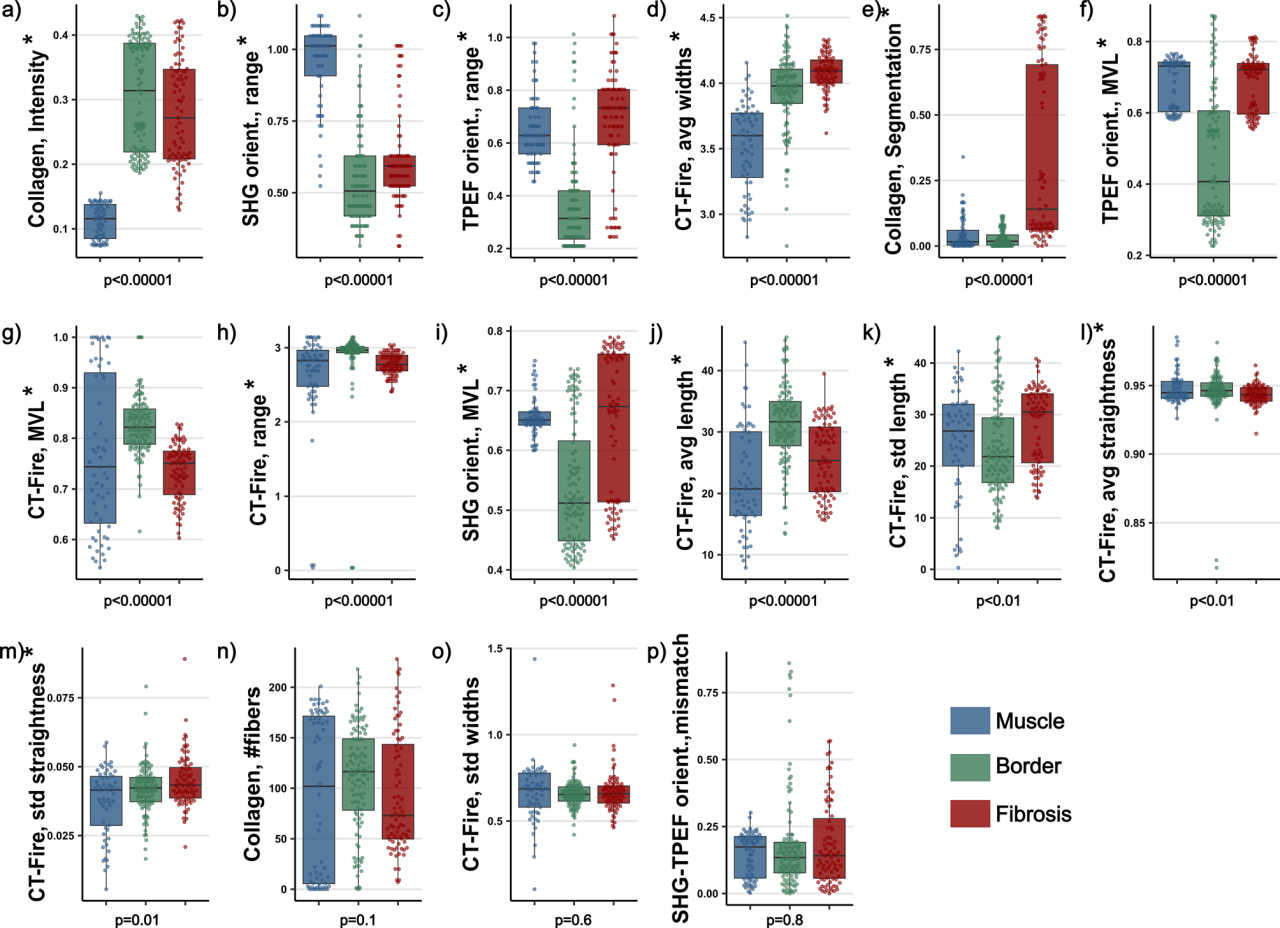
Boxplots of the 16 conventional imaging features in order from lowest to highest 3-class Kruskall-Wallis p value (**a**–**p**). The 3 classes are muscle (in blue, N = 67), border (in green, N = 112) and fibrosis (in red, N = 83). The features marked with a star (*) have a p value below 0.05 and deemed significant.

To quantify the qualitative differences between the classes, we extracted 16 conventional imaging features found in the literature from the images and tested their significance. A complete description of each feature including all mathematical definitions can be found in the methods section. These features can be classified into 3 groups: collagen amount, collagen fiber morphology and orientation features. Mean, standard deviation and p-value for each feature are found in Table 1.

**Table 1:** Mean and standard deviations for each of the 16 conventional imaging features and each of the 3 classes (Muscle N = 67, Border N = 112, Fibrotic N = 83).

Feature name	Muscle	Border	Fibrosis	p Value
	mean ± std	mean ± std	mean ± std	
Collange, Intensity*	0.11 ± 0.03	0.31 ± 0.08	0.28 ± 0.08	<0.00001
Collagen, Segment.*	0.04 ± 0.06	0.03 ± 0.03	0.34 ± 0.33	<0.00001
Collagen, # fibers	90.1 ± 78.8	109.6 ± 52	94.2 ± 58.8	0.14
CT-FIRE, avg lengths*	22.2 ± 8.64	31.3 ± 6.51	25.6 ± 6.02	<0.00001
CT-FIRE, std lengths*	24.5 ± 10.5	23.6 ± 9.00	28.0 ± 7.55	<0.01
CT-FIRE, avg widths*	3.52 ± 0.34	3.93 ± 0.29	4.08 ± 0.14	<0.00001
CT-FIRE, std widths	0.66 ± 0.18	0.66 ± 0.08	0.67 ± 0.13	0.6
CT-FIRE, avg straightness*	0.95 ± 0.01	0.95 ± 0.02	0.94 ± 0.01	<0.01
CT-FIRE, std straightness*	0.04 ± 0.01	0.04 ± 0.01	0.05 ± 0.01	0.01
CT-FIRE, MVL*	0.77 ± 0.15	0.83 ± 0.06	0.73 ± 0.06	<0.00001
CT-FIRE, range*	2.61 ± 0.66	2.88 ± 0.49	2.78 ± 0.15	<0.00001
SHG orient., MVL*	0.66 ± 0.03	0.54 ± 0.10	0.65 ± 0.12	<0.00001
SHG orient., range*	0.96 ± 0.14	0.55 ± 0.17	0.62 ± 0.17	<0.00001
TPEF orient., MVL*	0.68 ± 0.07	0.47 ± 0.19	0.69 ± 0.08	<0.00001
TPEF orient., range*	0.65 ± 0.13	0.36 ± 0.19	0.67 ± 0.22	<0.00001
SHG-TPEF, mismatch	0.14 ± 0.08	0.18 ± 0.19	0.18 ± 0.16	0.8

P value for the Kruskal-Wallis test. Features with p value < 0.05 are deemed significant and are marked with a star (*).

2 of the 3 collagen amount features are significant (Fig. 2). The SHG to TPEF intensity ratio was significantly lower in healthy muscle tissue (0.11$\pm \, 0.03$) compared to the other two classes (border 0.31$\pm 0.08$, fibrosis 0.28$\pm 0.08$). The SHG to TPEF segmentation volume ratio is significantly higher in fibrotic tissue (0.34$\pm 0.33$, muscle 0.04$\pm 0.06$, border 0.03$\pm 0.03$). The number of fibers counted by the CT-FIRE algorithm described in the methods section was not found to be a significant feature for any of the 3 classes.

5 of the 6 fiber morphology features are significant. The average fiber length is significantly higher in the border tissue (31.3 $\pm 6.51$ $\upmu$m, muscle 22.2 $\pm 8.64$ $\upmu$m, fibrosis $25.6\pm 6.02$ $\upmu$m). The average fiber width is significantly lower in healthy muscle (3.52 $\pm 0.34$ $\upmu$m, border 3.93 $\pm 0.29$ $\upmu$m, fibrosis 4.08 $\pm 0.14$ $\upmu$m). The standard deviation of fiber lengths is significantly higher in fibrotic tissue (28.0 $\pm 7.55$ $\upmu$m, muscle 24.5 $\pm 10.5$ $\upmu$m, border 23.6$\pm 9.00$ $\upmu$m). Both the average and standard deviation of fiber straightness distinguishes fibrotic tissue from the other classes however the amplitude of the effect is very small in both cases. The standard deviation of fiber widths is not significant.

6 of the 7 orientation features are significant. The mean vector length (MVL) of the CT-FIRE orientation histograms is significantly higher for the border tissue (0.83$\pm 0.06$, muscle 0.77$\pm 0.15$, fibrosis 0.73$\pm 0.06$). The range of the same histograms is significantly higher for the necrotic tissue (2.96$\pm 0.39$ rad, muscle 2.61$\pm 0.66$ rad, border 2.88$\pm 0.15$ rad). The MVL of the SHG orientation histograms is significantly lower for the border tissue (0.54$\pm 0.10$, muscle 0.66$\pm 0.03$, fibrosis 0.65$\pm 0.12$). The range of the same histograms is significantly higher for the healthy muscle (0.96$\pm 0.14$ rad, border 0.55$\pm 0.17$ rad, fibrosis 0.65$\pm 0.12$ rad). The MVL of the TPEF orientation histograms is significantly lower in border tissue (0.47$\pm 0.19$, muscle 0.68$\pm 0.07$, fibrosis 0.69$\pm 0.08$). The range of the same histograms is significantly lower in border tissue (0.36$\pm 0.19$ rad, muscle 0.65$\pm 0.13$, fibrosis 0.67$\pm 0.22$). The mean vector angle mismatch between the SHG and TPEF channel is not significant.

**Figure 3:**
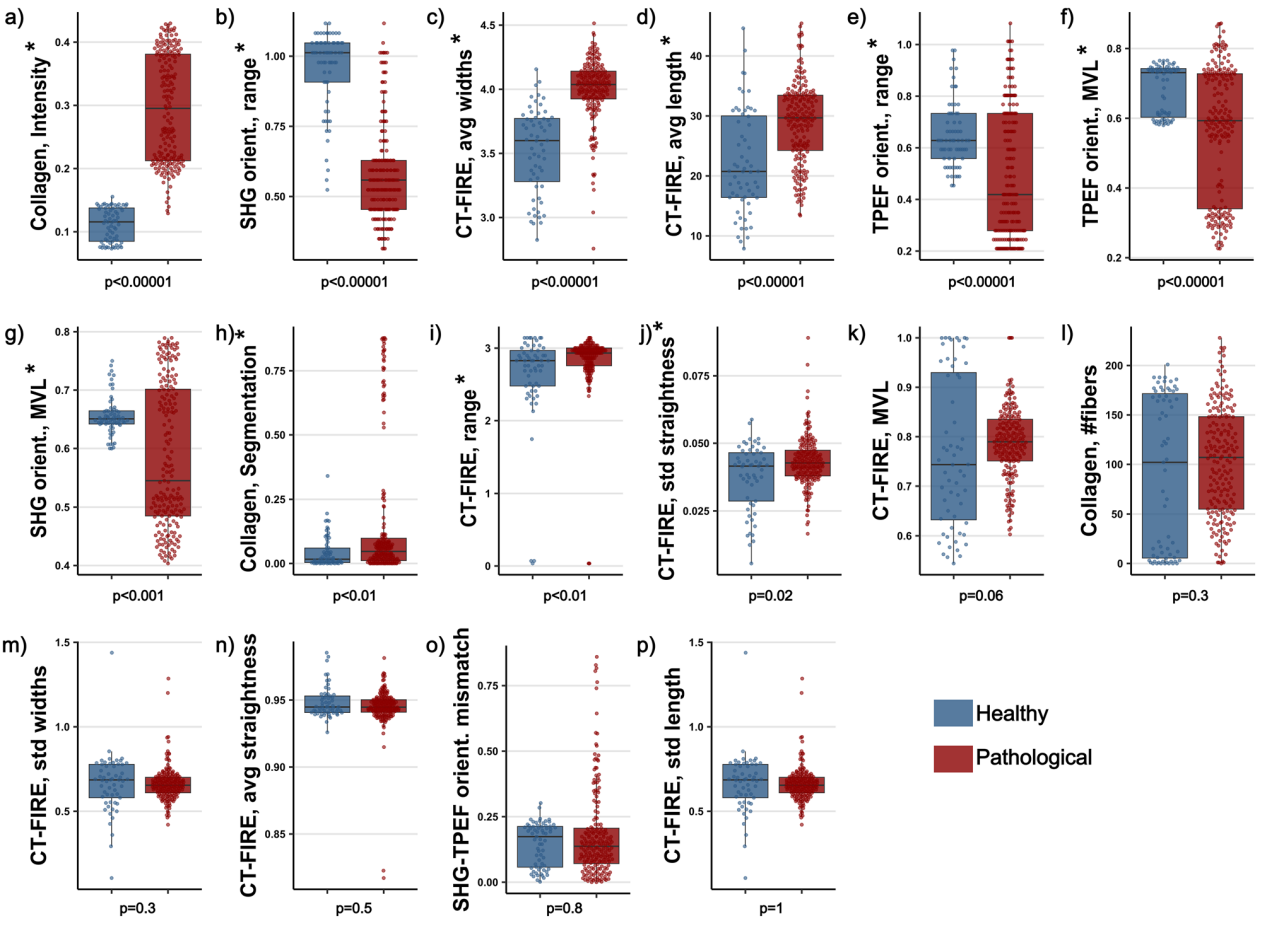
Boxplots of the 16 conventional imaging features in order from lowest to highest binary Wollaston p value (**a**)–(**p**). The 2 classes are healthy (in blue, N = 67) and pathological (in red, N = 195). The features marked with a star (*) have a p value below 0.05 and deemed significant.

Beyond the description of the different histological classes, the distinction between healthy muscle tissue and all types of pathological tissue is the most relevant for ablation surgery guidance. For this purpose, a binary test of each feature between healthy muscle tissue and all other classes (i.e. fibrotic and border combined) was conducted. 10 features were found to be significant (Fig. 3). Mean, standard deviation and p-value for each feature are found in Table 2.

**Table 2:** Mean and standard deviations for each of the 16 conventional imaging features comparing healthy muscle (N = 67) to the combined pathological states (N = 195). P value for the Wollaston ranksum test.

Feature name	Healthy	Pathological	p Value
	average ± std	average ± std	
Collagen, Intensity*	0.11 ± 0.03	0.30 ± 0.09	<0.00001
Collagen, Segmentation*	0.04 ± 0.06	0.16 ± 0.27	<0.01
Collagen, # fibers	90.1 ± 78.8	103 ± 55.3	0.3
CT-FIRE, avg length*	22.2 ± 8.64	28.9 ± 6.89	<0.00001
CT-FIRE, std length	24.5 ± 10.5	25.5 ± 8.67	1
CT-FIRE, avg widths*	3.52 ± 0.34	3.99 ± 0.25	<0.00001
CT-FIRE, std widths	0.66 ± 0.18	0.66 ± 0.10	0.3
CT-FIRE, avg straightness	0.95 ± 0.01	0.94 ± 0.02	0.5
CT-FIRE, std straightness*	0.04 ± 0.01	0.04 ± 0.01	0.02
CT-FIRE, MVL	0.77 ± 0.15	0.79 ± 0.07	0.06
CT-FIRE, range (rad)*	2.61 ± 0.66	2.84 ± 0.39	<0.01
SHG orientation, MVL*	0.66 ± 0.03	0.59 ± 0.12	<0.001
SHG orientation, range (rad)*	0.96 ± 0.14	0.58 ± 0.17	<0.00001
TPEF orientation, MVL*	0.68 ± 0.07	0.57 ± 0.19	<0.00001
TPEF orientation, range* (rad)	0.65 ± 0.13	0.49 ± 0.25	<0.00001
SHG-TPEF, mismatch (rad)	0.14 ± 0.08	0.18 ± 0.17	0.7

Features with p value < 0.05 are deemed significant and are marked with a star (*).

2 of the 3 collagen amount features are significant. The SHG to TPEF intensity ratio is significantly lower in healthy tissue (0.11 ± 0.03, pathological 0.30 ± 0.09). The SHG to TPEF segmentation volume ratio is significantly lower in healthy muscle tissue (0.04 ± 0.06, pathological 0.16 ± 0.27). The number of fibers extracted by the CT-FIRE algorithm is not significant.

3 of the 6 morphology features are significant. The average fiber length is significantly shorter in healthy tissue (22.2$\pm 8.64$ $\upmu$m, pathological 28.9$\pm 6.89$ $\upmu$m). The average fiber width is significantly thinner in healthy tissue (3.52$\pm 0.34$ $\upmu$m, pathological 3.99$\pm 0.25$ $\upmu$m). Although the standard deviation of the fibers’ straightness is significant, the strength of the effect is very small. The standard deviation of fibers lengths, the standard deviation of fibers widths and the average straightness of fibers are not significant.

5 of the 7 orientation features are significant. The range of the CT-FIRE orientation histograms is significantly lower in healthy tissue (2.61$\pm 0.66$ rad, pathological 2.84$\pm 0.39$ rad). The MVL of the SHG orientation histograms is significantly higher in healthy tissue (0.66$\pm 0.03$, pathological 0.59$\pm 0.12$). The range of the SHG orientation histograms is significantly larger in healthy muscle tissue (0.96$\pm 0.14$ rad, pathological 0.58$\pm 0.17$). The MVL of the TPEF orientation histograms is significantly larger in healthy muscle tissue (0.68$\pm 0.07$, pathological 0.57$\pm 0.19$). The range of the TPEF orientation histograms was significantly higher in the healthy muscle tissue (0.65$\pm 0.13$ rad, pathological 0.49$\pm 0.25$ rad). The range of the CT-FIRE orientation histogram as well as the mean vector angle mismatch between the SHG and TPEF channel are not significant.

### Radiomic features extraction, machine learning and quantitative comparison to conventional imaging features

152 Radiomic features were automatically extracted from each modality for a total of 304 feature. This initial dataset was pared down to the most quantitative features based on kernel density overlap, resulting in total of 26 radiomic features in the final dataset (14 SHG features, 12 TPEF features, see methods section for more details). This final dataset was used to train the AutoML algorithm which attributed a non-zero weight to 20 of the 26 radiomic features used for 3 class classification (Table 3). 9 of the 12 TPEF features received a non-zero weight with a highest feature weight of 9.99% ± 6.44% and an average combined weight of 43.8%. 11 of the 14 SHG features received a non-zero weight with a highest feature weight of 6.58% ± 8.27% and an average combined weight of 40.7%.

**Table 3:** Mean and standard deviation for the weight attributed by the machine learning to each of the 20 radiomic features with non-zero weight and the corresponding kernel density distribution overlap between the training and test sets for each.

Radiomic feature name	ranking weight	KDE overlap
	average ± dev	
TPEF::Histogram::ih.max	9.99% ± 6.44%	0.79
TPEF::GLSZM::szm.lgze	6.76% ± 6.63%	0.79
TPEF::Histogram::ih.max.grad	6.66% ± 4.48%	0.76
SHG::NGLDM::ngl.dc.entr	6.58% ± 8.27%	0.77
SHG::GLRLM::rlm.lrhge	6.42% ± 5.96%	0.76
TPEF::NGTDM::ntg.complexity	6.20% ± 4.11%	0.76
SHG::Intensity::stat.min	5.85% ± 3.98%	0.79
SHG::Intensity::stat.mean	5.04% ± 6.40%	0.76
SHG::IVHistogram::ivh.V90	4.73% ± 3.79%	0.84
TPEF::GLSZM::szm.zsnu	4.51% ± 3.82%	0.85
SHG::Intensity::loc.peak.global	4.51% ± 6.07%	0.83
SHG::Histogram::ih.min.grad.gl	4.47% ± 2.38%	0.78
TPEF::GLCM::cm.joint.max	4.05% ± 4.13%	0.79
TPEF::Morphological::morph.av	2.72% ± 1.95%	0.80
TPEF::GLCM::cm.inv.diff.mom.norm	2.39% ± 1.10%	0.80
SHG::GLCM::cm.inv.diff.mom.norm	1.88% ± 2.86%	0.79
SHG::GLSZM::szm.zs.entr	1.27% ± 2.23%	0.87
SHG::Morphological::morph.com	1.23% ± 1.63%	0.80
SHG::GLCM::cm.corr	0.63% ± 0.46%	0.75
TPEF::Histogram::ih.qcod	0.47% ± 0.47%	0.75

**Figure 4:**
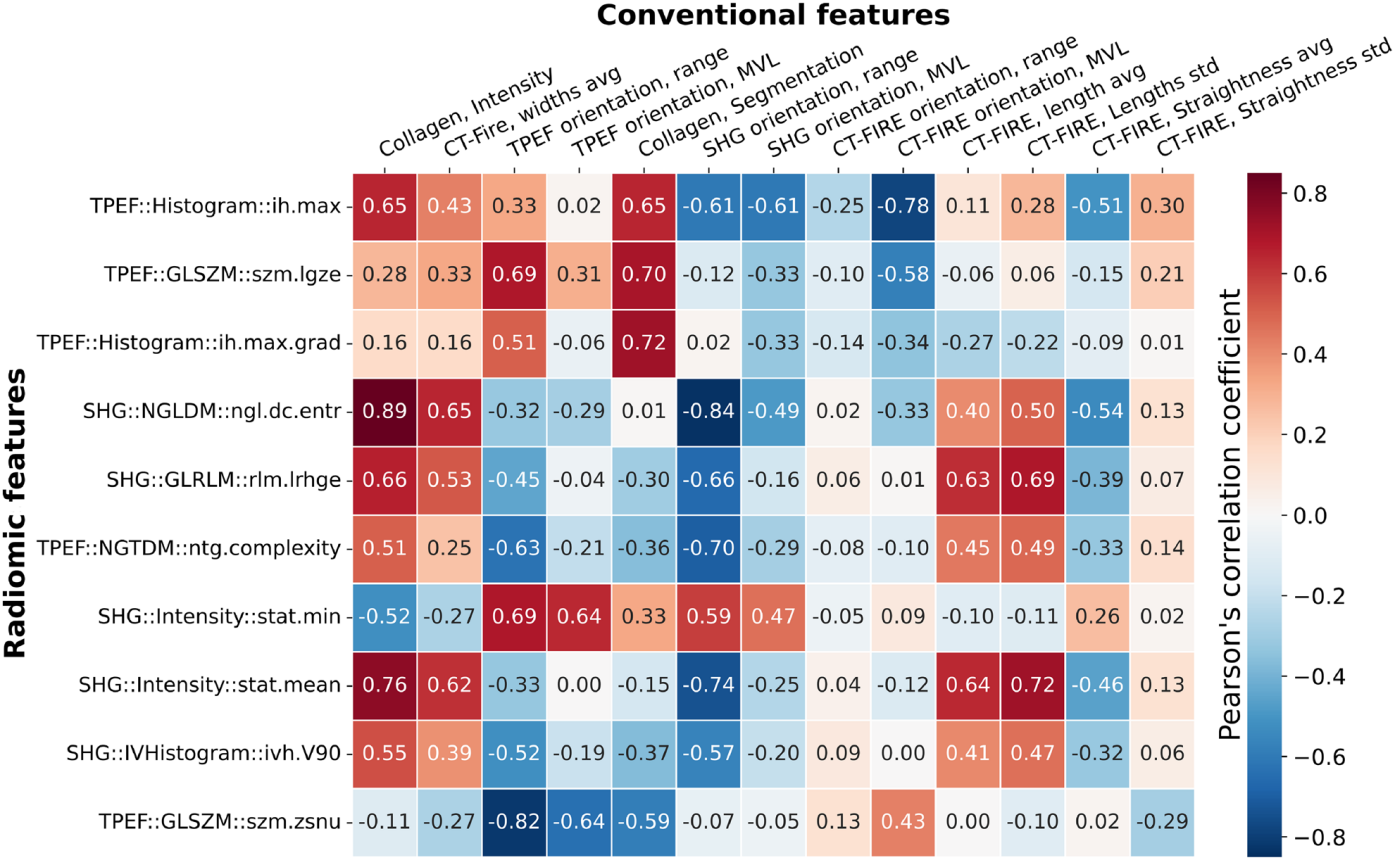
Heatmap of the Pearson correlation coefficients between the significant conventional features (on top) and the 10 most relevant radiomic features by weight (on the left). The features are classified from top to bottom with decreasing machine learning rank weight and from left to right with increasing Kruskall-Wallis p values.

To give biological context to the abstract radiomic features, we calculated the Pearson’s correlation between the radiomic features and the conventional features described in the previous section for the entire dataset. The results are shown as a heatmap in Fig. 4.

**Figure 5:**
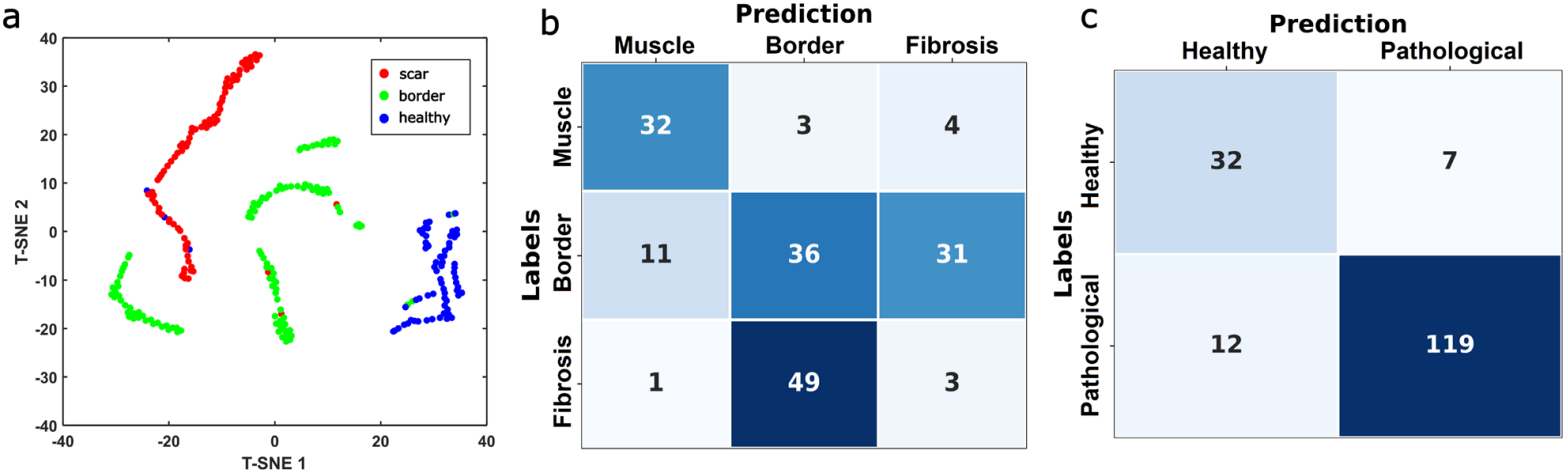
(**a**) T-distributed stochastic neighbor embedding (t-sne) map of the 26 relevant NLOM radiomic features, city-block distance, perplexity 15. Each dot represents an image with muscle in blue, border in green and fibrosis in red. Test set confusion matrices for the 3 class machine learning (**b**) and 2 class machine learning (**c**).

The 3 most prevalent radiomic features by weight are the TPEF maximum intensity of the histogram (histogram ih.max, weigth:$9.99\pm 6.44\%$), the TPEF low gray level zone emphasis for the gray level size zone matrix (GLSZM szm.lgze, weight:$6.76\pm 6.63\%$) and the TPEF maximum of the histogram’s gradient (histogram ih.max.grad, weight:$6.66\pm 4.48\%$). These features describe images with small regions of very high contrast and intensity in the TPEF channels surrounded by large area of low intensity. This can be linked to small pockets of surviving myocardium remaining inside the scar tissue. As can be seen in Fig. 4, these features share a positive correlation to the collagen intensity ratio (from 0.16 to 0.65), the collagen fibers average widths (from 0.16 to 0.43), the TPEF orientation range (from 0.33 to 0.69) and the collagen segmentation ratio (from 0.65 to 0.72). They also share a negative correlation to the SHG orientation MVL (from -0.33 to -0.61), the CT-FIRE orientation range (from -0.10 to -0.25) and the CT-FIRE orientation MVL (from -0.34 to -0.78). A group of 5 radiomic features share similar correlation coefficients to the same set of conventional features. The SHG counts entropy of the neighbouring gray level dependance matrix (NGLDM ngl.dc.ent., weight:$6.58\pm 8.27\%$) is a measure of overall randomness in the coarseness texture of an image. The SHG long run high gray level emphasis of the gray level run length matrix (GLRLM lrhge, weight:$6.42\pm 5.96\%$) is a measure of the abundance of long and high intensity fiber-like structures in an image. The TPEF complexity of the neighbouring gray tone difference matrix (NGTDM complexity, weight:$6.20\pm 4.11\%$) is a measure of the rapid, non-uniform changes of intensity in an image. The SHG mean intensity (Intensity stat.mean, weight:$5.04\pm 6.40\%$) represents the average intensity in the SHG channel. The SHG volume at $90\%$ intensity fraction (IVHistogram ivh.V90, weight:$4.73\pm 3.79\%$) is a measure of the proportion of the total number of pixels with an intensity equal or higher than $90\%$ of the maximum intensity. These features describe long, thick, intense, fiber-like objects in the SHG channel which correspond to the proliferation of collagen in fibrotic tissue. Together, they correlate positively to collagen intensity ratio (from 0.51 to 0.89), the collagen fibers average widths (from 0.25 to 0.65), the collagen fibers average length (from 0.40 to 0.64) and the collagen fibers standard deviation (from 0.47 to 0.64). Furthermore, they correlate negatively to orientation range in the TPEF channel (from − 0.32 to − 0.63), the orientation range in the SHG channel (from − 0.57 to − 0.84), the orientation mean vector length in the SHG channel (from − 0.16 to − 0.49) and the average straightness of collagen fibers (from − 0.32 to − 0.54). A 6th radiomic feature, the SHG minimum intensity value (Intensity stat.min) is inversely correlated to the same set of conventional features as the 5 features previously mentioned. As such, it can be understood as part of the same set of features, only describing the inverse of the other 5. Taken together, these 6 features are prevalent in the machine learning classifier results with a combined weight of $34.82\%$. The complete mathematical definition of each imaging feature can be found on the IBSI website^26^. Figure 5a presents a T-distributed stochastic neighbor embedding (T-sne) map of the 26 radiomics from the entire dataset.

The internal split for the AutoML service resulted in 100 train-validate subsets. Across the validate subsets, the validation predictive performance of the top-level mixed-stacked ensemble model was on average $95\pm 4.5\%$ for three-class classification. This performance was determined by confusion matrix analytics across all MC folds (see Supplementary Materials).

Subsequently, we applied the trained ensemble algorithm to the test set (170 total images) and compared the classification results from the algorithm to the labels. In the independent test set, the accuracy was 42% with the three-class classification (per-class sensitivities: 82% healthy, 46% border and 6% scar). In contrast the binary classification resulted in 89% accuracy (91% sensitivity for predicting pathological tissue and 82% specificity to predict healthy tissue).

The result of this independent test is presented on the confusion matrix in Fig. 5b. 32 out of the 39 muscle tissue images in the test set were correctly predicted. Out of the 78 images labeled as border in the test set 36 were correctly predicted to be border, 31 were erroneously predicted to be scar tissue and 11 were erroneously predicted to be healthy muscle tissue. Out of the 53 images labeled as scar tissue, 49 were erroneously classified as border by the algorithm, making it by far the most common error. This resulted in an overall 3 class accuracy of $42\%$, mostly caused by the errors in distinguishing between scar and border images. We also tested the binary accuracy of the algorithm by combining the fibrosis and border labels together into a “Pathological” label. This binary classification yielded the confusion matrix shown in Fig. 5c. 32 of the 39 muscle images were correctly classified and 119 out of 131 pathological images were correctly classified resulting in an overall binary accuracy of $89\%$.

Finally, to make the comparison between radiomic and conventional features as fair as possible, a second mixed-stacked ensemble learning model was trained on the 16 conventional features. The cross-validation accuracy was 85 ± 7% in the 92 images train-validation set. When applied to the 170 images independent test set, the 3-class accuracy was 56$\%$ (per-class sensitivity: healthy 73$\%$, border 86$\%$ and fibrosis 0$\%$). When combining the border and fibrosis labels together, the binary accuracy was 73$\%$. This was done to ensure that the conventional features did not contain relevant information hidden to the radiomics.

## Discussion

We explored the quantitative approaches for image analysis of SHG and TPEF found in the literature and extracted 16 conventional imaging features that describe the collagen amount, the fiber morphology and the directionality of the images. The collagen amount features denote an increase in and near the scar. 5 fiber morphology features significantly distinguish at least one of the classes which, overall, denote an increase in average fiber length and thickness in and near the scar. 6 of the orientation features significantly distinguish at least one of the classes. From these, the SHG orientation range is the one that most strongly distinguishes the healthy muscle from the others. For the purpose of ablation surgery guidance, a binary distinction between healthy muscle tissue and all pathological classes is required. We therefore tested the same set of 16 features combining border and fibrosis into a pathological class. Overall, these conventional imaging features agree with a qualitative description of the images: the scarring process is characterized by an increase in collagen amount, thicker and longer collagen fibers and a more scattered orientation both in the SHG and the TPEF channels. To our knowledge, this work is the first systematic approach to the application of these conventional imaging features on NLOM microscopy images of MI and as such we argue that it could serve as a first step towards systematizing and homogenizing the definitions used for conventional image analysis.

The most significant drawback of this conventional approach are the subjective and time consuming image pre-processing steps it requires that cannot all be automatized. This prevents the analysis of a big enough set of images that would be required for more advanced artificial intelligence techniques such as deep learning. In this work we showed that the use of IBSI radiomic features present a number of advantages over conventional image analysis for this type of images. Radiomics solve the problems of inconsistent definitions as well as time consuming and subjective pre-processing steps by establishing a robust set of mathematically consistent, quantitative and objective features that can be calculated automatically with comparatively little pre-processing of the image. We selected the 26 most quantitative independent radiomics subset to describe the images by calculating the kernel density estimation (KDE) overlap between the train and test set for each feature with a threshold of 0.75%. SHG and TPEF had similar levels of KDE overlap with respectively 14 and 12 features above threshold.

To give radiomics more biological context, we calculated the Pearson’s correlation between radiomics and conventional features. Among the 10 most significant radiomic features, we could identify a set of 3 TPEF based features describing small, very intense and contrasted regions that correlated with collagen amount, fiber thickness and multiple orientation features that correspond to scar tissue. We also identified a set of 6 radiomic features, 5 of which are based on SHG, that describe long, thick and curly collagen fibers as a marker for pathological tissue. On top of giving context to the radiomics feature, this correlation matrix shows that all 13 significant conventional features were correlated to at least 1 radiomics feature with a coefficient of at least 0.25, with 11 of them correlating to at least 1 radiomics feature with a coefficient of at least 0.50. We argue that the high correlation between the two sets of features shows that the radiomic features capture all of the most relevant biological information in the images in a systematic way. To our knowledge, this is the first time such systematic quantitative image analysis was operated on non-linear microscopy images of MI.

In order to demonstrate the usefulness of IBSI radiomic features for machine learning, we trained a mixed-stacked ensemble machine learning classification algorithm on a subset of 92 images. A 100-fold cross validation step yielded a 3 class accuracy of $95\pm 4.5\%$ within the validate subsets that were results of an internal Monte Carlo split. TPEF and SHG were deemed relevant with similar weights by the algorithm with a combined averaged weight of 43.8% and 40.7% respectively. We then performed independent testing of the algorithm on the remaining 170 images which yielded a $42\%$ accuracy for the 3 class classification. We believe that this drop in accuracy indicates a batch effect between the train and test sets, acquired from 2 different animals. Batch effects can stem from the fact that the underlying modalities, SHG and TPEF, are not completely quantitative. We accounted for this by harmonizing the features using the KDE overlap method but this drop in accuracy indicates that future research need to focus on the quantitative aspects of SHG and TPEF, for instance by integrating fluorescence lifetime imaging or applying novel image-level harmonization approaches based on generative adversarial networks, to fully unlock the potential of radiomics in the context of optical microscopy. The algorithm was notably better at identifying the healthy tissue (32/39 images) than fibrotic tissue (3/ 53 images) and border (36/78 images). The performances for identifying fibrotic tissue was notably poor. While there is a clear distinction between healthy tissue and all pathological classes, border and fibrotic regions are more difficult to distinguish from one another. Border ROIs in particular were very heterogeneous. Histology revealed a wide variety of tissue types: necrotic tissue, temporary granulation tissue and early stages of fibrosis. This was caused by the fact that scar formation and maturation happen at different rates in the infarcted area due to local differences in perfusion, inflammation and mechanical strain making the region inhomogeneous. To account for such heterogeneity we increased the number of border ROIs (4 instead of 2) in our dataset. Nevertheless, the binary classification showed promising results, especially considering the limited number of images. These results highlight the potential of our approach for binary discrimination between healthy and pathological tissue during surgical interventions. Further data collection would however be essential to enable the development of a robust and generalizable multi-class classification model.

The difficulty in reliably distinguishing border from fibrotic tissue reflects not only biological heterogeneity but also a deeper methodological challenge: the lack of standardized, high-resolution metrics that map onto mechanistic determinants of arrhythmogenicity. While features such as collagen fiber morphology, fiber orientation, and cardiomyocyte density provide a quantitative framework, additional parameters, such as the organization of residual viable myocardium into conduction channels, the persistence of sub-endocardial Purkinje fibers, or the presence of electromechanical decoupling zones, are known or hypothesized to contribute to arrhythmia vulnerability. These attributes are rarely captured comprehensively by existing imaging systems. Our approach, leveraging SHG and TPEF microscopy, enables visualization of several of these key micro-structural components, supporting the development of more precise classification schemes. However, current computational models remain limited in capturing the full complexity of these biological phenomena. Ongoing efforts are required to refine feature extraction pipelines, expand training datasets, and ultimately converge in a biologically grounded framework for scar classification that is predictive of electrophysiological behavior and arrhythmia risk.

To give conventional features a fair comparison, we trained a second mixed-stacked ensemble learning model. The model trained on conventional features showed cross-validation accuracy of 85$\pm 7\%$, a 56$\%$ 3-class accuracy in the independent test set and a binary accuracy of 76$\%$ in the independent test set. All in all, both models performed with comparable accuracies. This result, combined with the high level of correlation between the two sets, demonstrates that there is no significant information contained in the conventional features that is not accessible to the radiomics. Therefore, because of the time consuming and only partially automated pre-processing steps that the conventional features require, we argue that the automatically extracted radiomics are much better fitted for the dataset sizes required by machine learning.

In conclusion, the here presented work illustrates that the combination of SHG and TPEF microscopy based on endogenous contrast provides high resolution, sub-cellular scale images of myocardial infarcts. The images produced are comparable to the state-of-the-art histology techniques used such as Masson’s trichrome while requiring simpler, fewer and non-destructive preparation steps, no exogenous stains and the possibility to image tissue in depth. While most of the literature on non-linear optical microscopy for scar tissue focuses on the analysis of SHG imaging features, our work shows that the inclusion of TPEF as a second modality provides important complementary information of the relevant biomarkers for diagnosing and studying myocardial infarction at the cellular scale. Indeed, we found both SHG and TPEF based features to be significant by all methods used. Since the addition of TPEF to SHG is an inexpensive and straightforward step for imaging purposes and the image analysis of TPEF images uses the same tools as SHG images, we argue that its inclusion in future work on image analysis of non-linear optical microscopy of myocardial scar is an important step towards a better understanding of the scarring process. This work provides also systematic overview of the conventional image analysis methods used in the literature to quantify fibrosis in NLOM images and their respective significance on a real ex-vivo ovine infarction model. In parallel, it explores the potential for the application of standardized IBSI radiomic features as a tool to objectively and consistently quantify the images. The high correlation between these two sets of imaging features signals that there is no significant loss of information when using IBSI radiomic features over conventional features to describe the samples. Furthermore, the objective nature of IBSI radiomic features combined with the low amount of image processing needed to automatically extract them from a big image dataset make them ideal candidates for machine learning. Taken together, these results denote the potential use of IBSI radiomics as the basis for further work on MI image analysis and especially for automatic classification and diagnostic support. NLOM techniques have the potential to be integrated into endoscopic devices which could be used in the future to provide label-free, high-resolution and tissue-specific contrast of the myocardium in vivo. In particular, recent advances in femtosecond fiber lasers and micro-optical elements (GRIN lenses, MEMS scanners) are paving the way for miniaturizing and integrating NLOM into fiber-delivered, endoscopic systems. Our work demonstrates that images acquired with these devices could then be fed to an algorithm to guide ablation surgery with much higher spatial resolution compared to the current methods. The main drawbacks of endoscopic approaches are typically small field of views accessible with high numerical aperture (NA) lenses but recent advances in resonant scanning and adaptive sampling methods are steadily increasing the acquisition speed of endoscopic systems to counteract this limitation. Moreover, NLOM endoscopes could be used in complement to electro-anatomical mapping and CMR to take advantage of the increased resolution to find the critical isthmus for re-entrant tachyarrhythmia. Such a device would have the potential to make surgery less invasive and the ablation more precise leading to fewer MI recurrence and better patient outcomes.

## Methods

### Ovine myocardial infarction model and sample preparation

An ovine infarction model was provided by the Heart Rhythm Disease Institute, Bordeaux University Hospital, France in accordance with the guidelines from Directive 2010/63/EU – 2010 of the European Parliament on the protection of animals used for scientific purposes. All animal experiments were approved by the French Ministry of Higher Education and Research for the Care and Use of Experimental Animals. The present study was reviewed and approved by the local ethical committee at the University of Bordeaux (CEEA50). Following the 3Rs principles of reducing the number of animals used for research, this study was conducted in parallel with other experiments on the same tissue to minimize animal use.

The animals (N = 2) were 1 year old sheep (*Ovis aries*). The samples were produced following the methods described in details in^27,28^, of which the description included here is only a short summary.

**Figure 6:**
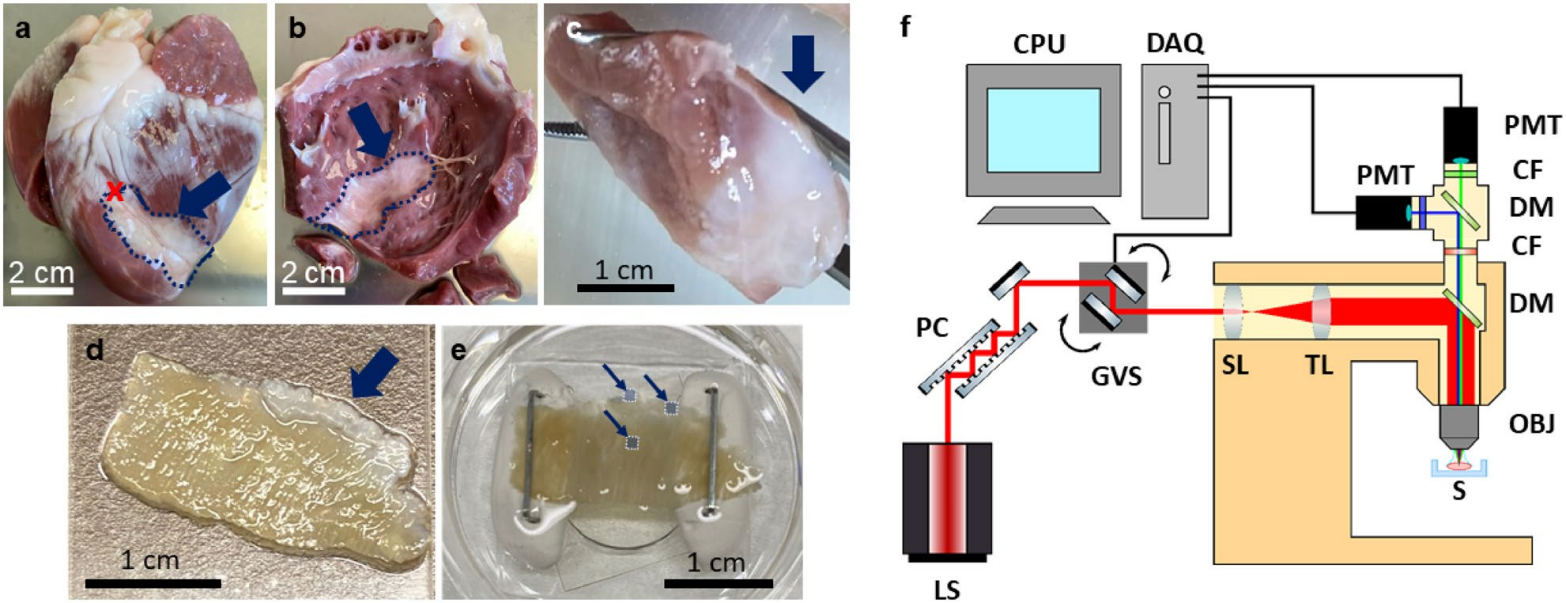
Images of the sample processing steps and non-linear optical microscope. (**a**) Picture of a harvested ovine heart, left ventricle facing up. A red cross marks the position of embolization on the left anterior descending coronary, approximately 8 cm up from the apex. The induced infarct in white is marked by a blue dashed line and arrow, around the artery distally from the embolization point. (**b**) Dissected left ventricle, inside view. The white transmural infarct is marked by a dark blue dashed line and arrow. (**c**) Myocardium chunk containing the infarcted tissue visible in white, designated by the dark blue arrow. (**d**) Fixated 400 $\upmu$m thick slice of myocardium. Visible white scar designated by the dark blue arrow. (**e**) Mounted slice in the microscopy dish, stapled into the putty for stability. 0.5 mm by 0.5 mm ROIs at and around the scar are marked by light blue squares and designated by dark blue arrows. (**f**) Diagram of the custom-built multimodal non-linear microscope. LS: NLOM laser, PC: precompensation mirrors, GVS: Galvo scanners, SL: scan lens, TL: tube lens, DM: dichroic mirror, OBJ: objective, S: sample, CF: chromatic filter, PMT: photomultiplier tube, DAQ: data acquisition card, CPU: computer.

The animals underwent an initial surgery under general anesthesia to induce MI. A catheter was introduced into the femoral artery and advanced through the aorta to the left coronary artery, located on the left ventricle. Infarction was triggered by placing an embolization coil in the left anterior descending artery, approximately 8 cm from the heart’s apex. Following the procedure, the animals were allowed to recover in accordance with standard protocols and were monitored for a 6 week recovery period. During this time, scarring developed in the ischemic region of the left ventricle. Figure 6a depicts an ovine heart, highlighting the white scar on the left ventricle and the embolization site. The scar tissue formed around the coronary artery downstream of the embolization site.

After 6 weeks of recovery, the animals were euthanized by intravenous injection with pentobarbital (30 mL/50 Kg) and their hearts were harvested. Figure 6a–e illustrate the steps for sample preparation. Initially, the left ventricle was separated from the harvested hearts (Fig. 6b). The area surrounding the infarct scar was then sectioned into chunks measuring approximately 2 cm by 2 cm (Fig. 6c). These chunks were further sliced into 400 $\upmu$m-thick transverse sections, creating trans-mural slices. The slices were subsequently dehydrated and fixed using ethanol (Fig. 6d). After transport, the slices were mounted onto microscope dishes with putty and staples for stabilization (Fig. 6e) and immersed in water to support imaging with a water immersion objective. Finally, ROIs within the scar, in the healthy myocardium and at the border were designated for analysis using multimodal non-linear optical microscopy. In the ovine myocardial infarction model, the infarct border is typically evident by a sharp visual transition from healthy myocardium to discolored or texturally altered tissue. To avoid misclassification, particularly distinguishing scar from native structures such as epicardial fat – border delineation was always guided and validated by experienced heart-biology specialists familiar with infarct anatomy in this model and later confirmed with histology. Once the boundaries were identified, multiple ROIs were selected along the border zone to capture a representative range of tissue morphology and microstructural heterogeneity.

### Multimodal non-linear optical microscopy

The multimodal imaging system including SHG and TPEF microscopy has been previously described in details in^25^. The system is based on a custom-built Kerr-lens mode-locked ultrashort Titanium Sapphire oscillator operating at 800 nm central wavelength with a spectral bandwidth at full-width-half-maximum of 15 nm. The average power at the sample was set to 80 mW, corresponding to 1.1 nJ pulses at a repetition rate of 72.2 MHz, and kept it constant for all images. The system includes a pair of negative chirp mirrors (DCMP175, Thorlabs GmbH, Germany) on the beam path to compensate the dispersion caused by the objective and the other optics to guarantee a sub 70 fs pulse duration at the sample plane.

The images were acquired using a customized laser scanning microscope based on a Nikon microscope frame (Eclipse E400, Nikon, Tokyo, Japan. Figure 6 f. A pair of galvanometric scanning mirrors (6230H, Cambridge Technology, Bedford, MA, USA), a scan lens (AC254-050-B, Thorlabs GmbH, Germany) and a tube lens (AC254-200-B, Thorlabs GmbH, Germany) generate the raster scanning pattern on the sample through a 16x, 0.8 NA water immersion objective (CFI LWD Plan Fluorite Objective, Nikon, Japan).

Both modalities were collected in epi direction through the same objective. A dichroic mirror (T680SPXR, Chroma Technology GmbH, Germany) with a cut-on wavelength of 680 nm separates the laser from the signals. The signals were further cleaned up with a 750 nm low pass filter (FES0750, Thorlabs GmbH, Germany). The SHG and TPEF signals were separated using a long pass dichroic filter with a cut wavelength at 495 nm (T495LPXR, Chroma Technology GmbH, Germany). Each signal went through a chromatic filter (for TPEF: ET550/49 BrightLine HC, F37-446, Semrock Rochester, NY, USA, for SHG: ET405/10x BrightLine, Semrock Rochester, NY, USA) and was detected by a photomultiplier tube (H10723-01, Hamamatsu Photonics K. K., Japan). Gain on the detectors was kept constant for all images. The simultaneous excitation and collection of the SHG and TPEF signals was controlled through the open source software ScanImage^29^. A 1 $\upmu$m lateral and 3 $\upmu$m axial optical resolution was achieved with both NLOM modalities. The multimodal images acquired are 512 by 512 pixels and correspond to a 508 $\upmu$m by 508 $\upmu$m field of view. The frame rate was 0.83 Hz with 3.2 $\upmu$s pixel dwell time. Each image was averaged 20 times for a total acquisition time of 24 s per image.

**Figure 7:**
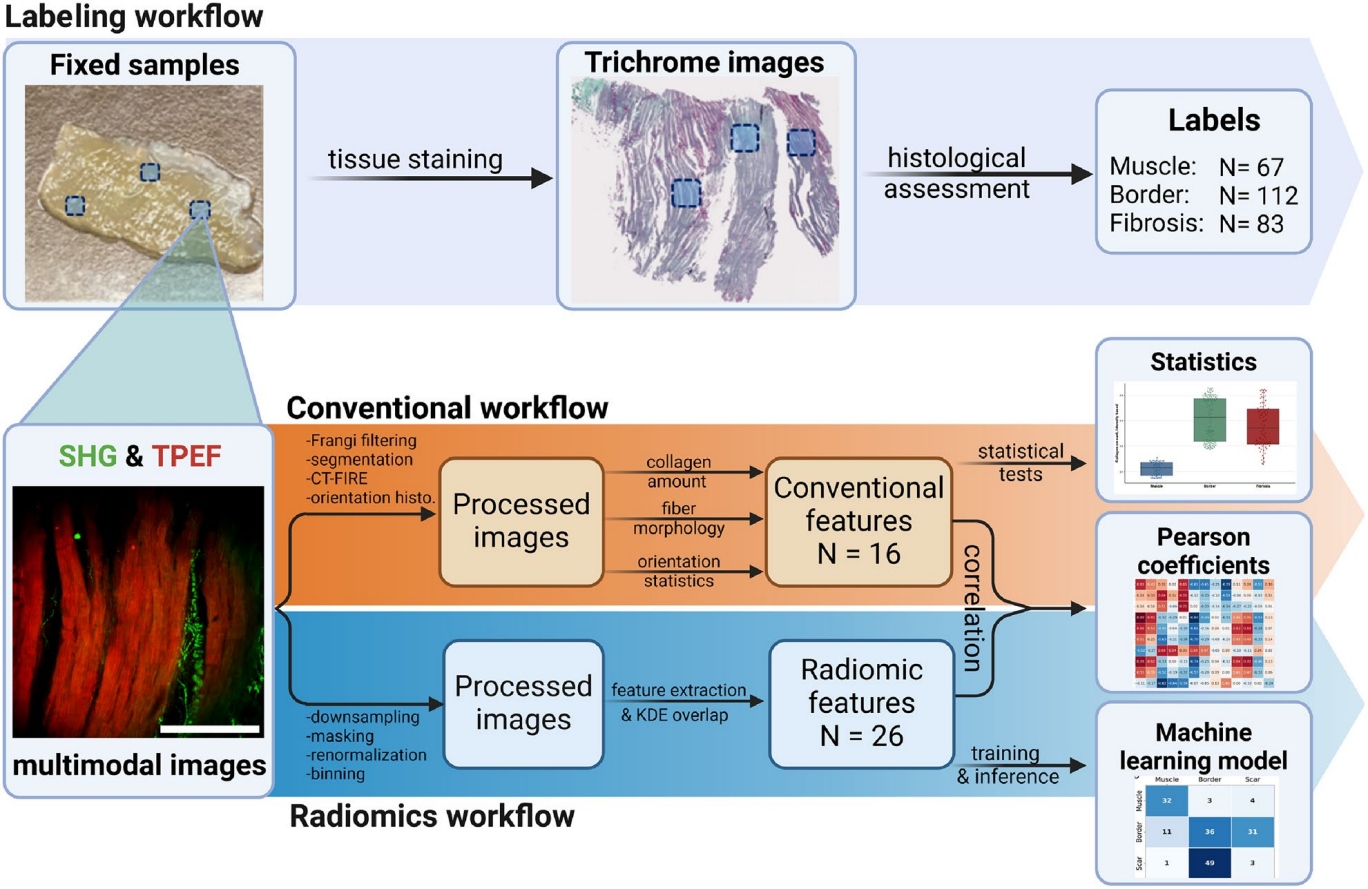
Overview of the image acquisition and analysis workflow. The ROIs (not to scale) are marked in light blue and localized on the trichrome stained images for labeling. Each ROI is imaged in depth with 5$\upmu$m steps starting from the surface. The multimodal NLOM images are then pre-processed following the workflow described in the method to extract 16 conventional features and 26 radiomic features. A correlation matrix is calculated between. Conventional features are submitted to statistical tests to ascertain their significance. Radiomic features are used to train a mixed-stacked ensemble learning model.

From the 3 myocardium samples, 8 relevant ROIs had been previously identified and marked. At each ROI, the surface was imaged first and then subsequent images were acquired at 5 $\upmu$m intervals in depth by translating the sample using a micrometer stage. The step size was chosen to be significantly bigger than the 3 $\upmu$m depth of field, guaranteeing that adjacent images are optically independent from each-other, while maximizing sampling of the tissue. Imaging could be achieved down to between 135 and 250 $\upmu$m below the tissue surface depending on the strength of the signal at a given ROI, corresponding to 26 to 51 images per ROI. This depth range was intentionally chosen to span multiple histologically distinct layers of the ventricular wall. Given that individual cardiomyocytes measure approximately 20 $\upmu$m in thickness and are organized into laminar myofiber sheets that change orientation and density with depth, this protocol enabled the capture of structurally diverse zones within each image stack. As such, the upper and lower regions of the stacks often revealed distinct morphological characteristics, consistent with transitions across myocardial strata and depth-dependent remodeling patterns observed in infarcted tissue. This imaging strategy ensures that the acquired data encompass a biologically relevant microenvironment and capture spatial heterogeneity critical for downstream image analysis and classification tasks. The depths achieved were approximately similar in all samples (min 135 $\upmu$m, max 250 $\upmu$m, mean 165 $\upmu$m). This allowed the potential effects of depth to be more or less equally distributed in each group. The images were saved in a 2 channels, 16 bits, multi-page Tif stack for each ROI. An overview of the entire image acquisition and analysis workflow is presented in Fig. 7.

### Masson’s trichrome and bright field optical microscopy

Following multimodal NLOM imaging, the tissue sections (approx. 400 $\upmu$m thick) were embedded in paraffin and serially sectioned at 2 $\upmu$m thickness using a vibratome. These thin slices were stained using a standard Masson’s trichrome protocol, highlighting collagen (green), muscle (pink), and nuclei (purple). Because the entire in-plane cross-section of each tissue sample was preserved on the microscope slide, ROI locations previously imaged by NLOM could be reliably identified by aligning tissue edge distances and common anatomical features across both modalities. Additional matching was performed based on morphological landmarks such as trabecular patterns or vascular remnants visible in both datasets. This permitted robust assignment of each ROI to one of three histological classes, healthy myocardium, scar border, or mature fibrosis, based on spatial context and local tissue composition. Bright field microscopy images of the slices were acquired using an inverted brightfield microscope (Thunder, Leica Microsystems, France). A trained pathologist confirmed the classification each of the 8 ROI into 3 categories: 2 ROIs were identified as healthy muscle, 2 as fibrotic tissue and 4 as border regions between healthy and fibrotic. This assessment based on the Masson’s trichrome stained images was used as ground truth for all further analysis.

### Conventional imaging features

The literature generally agrees on 3 main markers to distinguish between physiological and scar tissue in the case of the post-MI scarring process: collagen amount, collagen fibers morphology and directionality^7,13,14,19,30,31^. However, there is no consensus on the methods used to quantify each of these markers nor on the weight to give each of them. From the literature, we identified 3 features describing the collagen amount, 6 features describing the collagen fibers morphology and 7 features describing the directionality for a total of 16 conventional imaging features.

#### Collagen amount

The most straightforward method to quantify the collagen amount relative to myocardium on a given image is to calculate the ratio of average intensities of the SHG and TPEF channels as described in equation 1:

1 $$\begin{aligned} C_I = \frac{\overline{I_{SHG}}}{\overline{I_{SHG}} + \overline{I_{TPEF}}} \end{aligned}$$

where $\overline{I_{SHG}}$ and $\overline{I_{TPEF}}$ are the intensity values in the SHG channel and in the TPEF channel averaged over a given image. This was computed for each image using a custom Matlab script.

Another method to quantify the proportion of collagen in a given image is to segment the TPEF and SHG channels and calculate the ratio of segmented SHG and TPEF pixels as described in equation 2:

2 $$\begin{aligned} C_S =\frac{N_{SHG}}{N_{SHG} + N_{TPEF}} \end{aligned}$$

where $N_{SHG}$ and $N_{TPEF}$ are the total numbers of segmented pixels from the SHG and the TPEF channels in a given image.

$N_{SHG}$ and $N_{TPEF}$ were each calculated using the open source software ImageJ and its integrated Auto Threshold plug-in^32^. The TPEF and SHG channels were first separated resulting in a multi-page Tifs stack for each modality. For the TPEF modality, each stack was first converted from 10 to 8 bit with a linear rescaling between the maximum and minimum values of the entire stack. After that, no further processing was necessary and the threshold value was calculated using the maximum Rényi entropy algorithm with the entire stack as input, resulting in a binary stack^33^. For the SHG modality, some intermediary processing steps were required to enhance the contrast of collagen fibers against the background. Each slice was passed through a multi scale Frangi filter (3 scales at 1.8, 4.9, 8 pixels Gaussian diameter kernel). This was done using the Frangi Vesselness plug-in in ImageJ and resulted in 32 bit Frangi filtered images^34^. The images were then converted to 16 bit with linear rescaling over the entire stack. A threshold value was then calculated using the maximum Rényi entropy algorithm on the entire stack, resulting in a binary stack. Using these two binary stacks, the collagen proportion was calculated for each slice by adding up the pixels and computing the ratio shown in equation 2.

The third method to quantify collagen amount is the fiber count based on the CT-FIRE algorithm described in the next section.

#### Collagen fibers morphology

The CT-FIRE algorithm is the state-of-the-art for automatic measurement of collagen fiber morphology on SHG images. It is based on the fast discreet curvelet transform and the fiber extraction algorithm presented in^20^ and^35^ and the Matlab implementation they provide was used in the present work.

The processing steps are similar to the previous section. On the SHG stacks, the contrast of collagen fibers was enhanced using a multiscale Frangi filter (3 scales at 1.8, 4.9 and 8 pixels Gaussian diameter kernel). The images were then converted to 16 bits with linear rescaling over the entire stack.

Each Frangi filtered stack was then run through the CT-FIRE algorithm. The algorithm isolated the individual collagen fibers on each slice and computed the morphological features of each fiber to generate 3 histograms for each image: fiber length, fiber width and fiber straightness. The mean and the standard deviation were computed on these histograms leading to 6 morphological feature for each image.

#### Directionality

The spatial interlacing of collagen fibers is a fundamental characteristic of scar tissue. In the present work, we based our description on circular statistics as detailed in^36,37^. Circular statistics are calculated on a directionality histogram that must first be extracted from the images. This was done using the algorithm described in^31^ and implemented in the Directionality plug-in in ImageJ. This was done separately for the TPEF and the SHG channel. The output of this algorithm is a circular histogram going from 0$^{\circ }$ to 180$^{\circ }$ (1$^{\circ }$ bins) describing the general directionality of the image for each slice and each modality. In addition, the CT-FIRE algorithm described in the previous subsection also outputs a directionality histogram from the angles of each individual collagen fiber. In total, this yielded 3 directionality histograms per image: one from the TPEF channel, one from the SHG channel and one from the CT-FIRE algorithm.

From each of these histograms, the mean vector $\overline{V} = (\overline{X},\overline{Y})$ was calculated as follows:

3 $$\begin{aligned} \overline{X} =\sum _{\theta =0}^{\pi } f(\theta )cos(\theta ) \hspace{4mm},\hspace{4mm} \overline{Y} =\sum _{\theta =0}^{\pi } f(\theta )sin(\theta ) \end{aligned}$$

where $f(\theta )$ is the normalized frequency of the histogram for the angle value $\theta$, i.e $\sum _{\theta = 0}^{\pi } f(\theta ) = 1$ for each histogram.

The first quantitative measurement of angular dispersion is the mean vector length (MVL), calculated as $MVL= \sqrt{\overline{X}^2 + \overline{Y}^2}$. Because $f(\theta )$ is normalized, the MVL must by definition be a number between 0 (weak directionality) and 1 (strong directionality).

A complementary measure of angular dispersion is the range *R*, defined as the smallest arc containing half of the histogram’s total weight:

4 $$\begin{aligned} R = min_{k,m} (||\theta _k - \theta _m||) \hspace{4mm} \ni \hspace{4mm} \sum _{\alpha = \theta _k}^{\theta _m} f(\alpha ) > \frac{1}{2} \end{aligned}$$

The MVL and range were calculated from each of the 3 histograms resulting in 6 features per image. This was implemented using a custom script in Matlab, making sure to consider the periodicity of the histogram.

The 7th and last feature calculated was the average angle mismatch $\overline{M}$ between the SHG and TPEF channel as follows:

5 $$\begin{aligned} \overline{\Theta } = \arctan (\frac{\overline{X}}{\overline{Y}}) \hspace{4mm} , \hspace{4mm} \overline{M} = ||\overline{\Theta }_{SHG} - \overline{\Theta }_{TPEF} || \end{aligned}$$

#### Statistical analysis

A non-parametric 4 class Kruskall-Wallis ranksum test was applied to each of these 16 conventional imaging markers to check the significance of each without making any assumption on the normality of the distributions. Features with a p value below 0.05 were deemed significant.

Subsequently, a binary comparison between healthy muscle and all other pathological states combined was conducted by applying a non-parametric Wollaston ranksum test to each feature without making any assumption on the normality of the distributions. Features with a p value below 0.05 were deemed significant.

### Radiomics feature extraction and machine learning

To extract the IBSI radiomic features, each image was first binned down from 512 $\times$512 to 128$\times$128 pixels (4$\times$4 pixel bin size and averaging) according to best practices outlined in^38^. This was done to reduce the influence of noise and high-spatial-frequency components, which would dominate the radiomics of non-downsampled images. The size of the pixel (4$\times$4 $\upmu m^2$) was chosen as it is comparable to the thickness of a collagen fiber, guaranteeing minimum loss of relevant spatial information. A foreground mask was then automatically segmented from each modality using ImageJ’s Auto-Threshold plug-in with Li’s algorithm and combined with an OR operation. To result in semi-quantitative radiomic features, a renormalization step was used to smooth out the individual differences in relative intensities. For each image and each modality, the average intensity value of the background, i.e the pixels outside the foreground mask, was computed. The intensity values of each image were then divided by the average value of their corresponding background. The resulting renormalized images were then binned down to 8 bit using a linear rescaling over all images from all ROIs. This pre-processing resulted in 128$\times$128 pixels, 8 bit images for each modality (TPEF and SHG) and 1 binary 128$\times$ 128 pixels foreground mask.

Out of the 8 ROIs imaged, 3 were arbitrarily selected as training set (from subject #1, 1 healthy muscle, 1 fibrosis, 1 border) and the 5 remaining ROIs assigned to the independent test set (from subject #2, 1 healthy, 1 fibrosis, 3 border). This process resulted in a total of 92 images that served as training set and 170 images that served as independent test set. We purposefully segregated the 2 animals into the 2 subsets; subject #1 into the training set and subject #2 into the test set. This was done to guarantee independent validation and to demonstrate the generalizability of our approach beyond the training data.

The MUW Radiomics Engine ver. 2.0 was used to extract a total of 152 radiomic features from each multimodal image in both training and test sets^39^. A complete description of each of the 152 radiomic features and their detailed mathematical definitions can be found on the IBSI website^26^. Additionally, co-registered optical coherence tomography en-face images of the tissue yielded 132 radiomic features, however, the modality was found to be less descriptive than the other modalities involved and a more complete description of it was relegated to the supplementary materials. This step resulted in a total of 304 NLOM radiomic features per image. In order to select the most quantitative radiomic features for further analysis, we performed a kernel density estimation (KDE) in Matlab using the functions described in^40^. The KDE overlap between training and test sets was then calculated for each feature. A threshold of 0.75 KDE overlap was selected and radiomic features below this value were discarded resulting in 26 radiomic features of which 12 were based on TPEF and 14 were based on SHG.

Mixed-stacked ensemble learning was utilized to build 100 model instances across a 100-fold Monte Carlo (MC) train-validate split over the training set. This was performed by relying on the Dedicaid AutoML service (Telix Pharmaceuticals Ltd) hosted at the Medical University of Vienna^41,42^. In each MC fold, 20% of the cases were randomly selected to serve as internal validate set. The validate set was balanced across the given subgroups to predict, thus, leaving the train split imbalanced. The AutoML approach conducted an automated data pre-processing including feature range normalization, redundancy analysis and reduction as well as feature selection and class imbalance correction with synthetic training data points generation. This was followed by building a two-layer mixed, stacked machine learning as described in^41,42^. Automated Machine Learning (AutoML) was used to automatically select, optimize, and evaluate machine learning models and preprocessing pipelines for establishing classification model and to minimize manual intervention and bias. AutoML ensures an unbiased, reproducible, and efficient model development process by systematically exploring a wide range of algorithms and hyperparameter configurations. Besides minimizing manual trial-and-error, it reduces the risk of overfitting due to subjective operator bias, and enables the identification of optimal pipelines tailored to the data. A comprehensive description of the model is available in the supplementary materials.

Once all the 100 mixed-stacked ensemble model instances were built, inference was conducted over the independent test set samples. Here, a three-class confusion matrix was extracted to estimate the predictive performance of the ensemble model. The three-class confusion matrix was also collapsed to a binary classification confusion matrix, where the borderline and scar samples were categorized as pathological.

Finally, to make the comparison between radiomic and conventional features as fair as possible, a second mixed-stacked ensemble model based on the 16 conventional features was built following the same architecture as the radiomics based model.

### Comparison of radiomic and conventional features

We calculated the 16 conventional imaging features described in Sect. 2.4 on each of the 262 NLOM images acquired (train-test sets combined).

In order to identify IBSI-standardized radiomic features that are potential surrogates of conventional ones, quantitative comparison between conventional and radiomic features was performed. This step included the 13 most significant conventional imaging features and the 10 highest-ranking radiomic features based on the feature ranking of the AutoML service.

### Code availability

The algorithms used to calculate the conventional features are described in details in the present methods section. The various Matlab and FIJI scripts used are maintained at the Medical University of Vienna and are available upon reasonable request from the corresponding author. They are based in part on 3rd party software (Matlab and FIJI plug-ins, the CT-FIRE algorithm) which are maintained by their respective developers and accessible open-source^20,29,31,33–37^. For the radiomics extraction, the MUW Radiomics Engine was used, of which the underlying algorithms are public^39^. The engine is hosted and maintained at the Medical University of Vienna and accessible upon reasonable request from L.P.. The machine learning was based on the use of 3rd-party software AutoML^41,42^, a service of Dedicaid GmbH a wholly owned subsidary of Telix Pharmaceuticals Limited hosted at the Medical University of Vienna.

## Supplementary Information

Supplementary Information.

## Data Availability

The images underlying the results presented in this paper are not publicly available at this time but may be obtained from the authors upon reasonable request to the corresponding author, Angelika Unterhuber.
