# Supplementary material for: Comparison of conventional and radiomics-based analysis of myocardial infarction using multimodal non-linear optical microscopy
Source: Sci Rep. 2025 Jul 18;15:26040. doi: 10.1038/s41598-025-10515-y (PMC12271366; doi:10.1038/s41598-025-10515-y)
Supplement: Supplementary file 1 — Supplementary Information. [file 41598_2025_10515_MOESM1_ESM.pdf]

Supplementary material

TABLE OF CONTENTS

PREAMBLE 3
100-FOLD RADIOMICS-BASED AUTOML 3
AUTOMATED MACHINE LEARNING ANALYSIS 3
METHODS 4
Data 4
Notes on the OCT modality 4
Cross-Validation 6
Preprocessing 6
Machine Learning Layer 1 6
Machine Learning Layer 2 7
Top-Layer Model 7
RESULTS 8
Cross-Validation Performance 8
Feature Importance 9
References 13
100-FOLD CONVENTIONAL-BASED AUTOML 15
AUTOMATED MACHINE LEARNING ANALYSIS 15
METHODS 15
Data 15
Cross-Validation 16
Preprocessing 16
Machine Learning Layer 1 17
Machine Learning Layer 2 17
Top-Layer Model 18
RESULTS 18
Cross-Validation Performance 18
Feature Importance 20
References 22

PREAMBLE

This preamble shortly summarizes the automated machine learning (AutoML) algorithm used in this study.
AutoML is a fully-automated 100-fold mixed-stacked ensemble learning algorithm comprised of 3 layers: the first layer is a 100-fold primary machine-learning. Each fold is a unique, randomly assigned 80%/20% split of the training set. In each Monte-Carlo fold, one of three distinct machine-learning algorithms were used independently: Multi-Gaussian Weighted (MGWC, 28 folds) classifier, Random Forest (RF, 43 folds) classifier and Support Vector Machine (SVM, 29 folds) classifier. This is done to minimize the effect of algorithm bias. Then a meta-training set is created by evaluating each of the 100 ML algorithms of Layer 1 on each of the other Monte-Carlo folds' training set, resulting in 100^100 prediction results. These predictions are treated as meta-features. 100 Monte-Carlo folds of these meta-features are then randomly created (80%/20% random train/validation split). A second layer of 100 MGWC classifiers were then trained on these meta-features. This resulted in 100 mixed ensemble "super-learners". Finally, the top layer combines the predictions of the second layer models by the weighted majority vote to provide the final prediction. This results in a robust mixed-stacked ensemble learning model. This is the final result of the AutoML algorithm."

100-FOLD RADIOMICS-BASED AutoML

AUTOMATED MACHINE LEARNING ANALYSIS

Tabular data submission for Dedicaid[1] AutoML services was performed by user laszlo.papp@meduniwien.ac.at on 5/2/2024, 10:34:57 AM to build and cross-validate automated data preprocessing and mixed, stacked ensemble learning pipelines for predicting reference label Label. For the details of the analysis see Table 1.

Table 1. Properties of the automated machine learning (AutoML) analysis of this study.

Data name	TrainF-OCT-healthy-vs-B-vs-S-KDEordered-0.75KDECutoff.xlsx
Data size	92 samples, 85 features
Date of analysis	5/2/2024, 10:34:57 AM
Duration of analysis	11h 28m 17s
Submitted by	laszlo.papp@meduniwien.ac.at
Dedicaid AutoML version	0.1

[1]Dedicaid GmbH, a wholly owned subsidary of Telix Pharmaceuticals Limited

METHODS

Data

The input dataset was composed of 92 samples and 436 features from 3 modalities: second harmonic generation (label: SHG, 152 features), two-photon excited fluorescence (label: TPEF, 152 features), optical coherence tomography (label: OCT, 132 features).

To keep only the most quantitative features, an intermediary feature selection step was conducted. The selection was based on the kernel density overlap of the individual features between training and independent test sets was conducted with a threshold of 0.75 (see main text Methods section). This resulted in a submitted dataset composed of 85 features: 59 from the OCT modality, 14 from the SHG modality and 12 from the TPEF modality. The selected reference label for the cross-validation was Label having subgroups of missing (32.6%) label outcomes.

Notes on the OCT modality

OCT is a non-invasive optical imaging technique based on low coherence interferometry which provides morphological images in depth. In this multimodal imaging system, the OCT modality is based on a custom broadband Titanium Sapphire oscillator with 800 nm central wavelength and 140 nm full-width half maximum spectral bandwidth. For OCT, the power at the sample plane was set to 2.5 mW. The beams coming back from the sample and reference arms were combined in a fiber-based 80:20 beam splitter and the interference pattern was detected with a custom built spectrometer consisting of a 1200 l/mm grating (Wasatch Photonics, Logan, UT, USA) and a 2048 pixels, 12 bit CCD line-scan camera (AViiVA Atmel EM4CL 2014, Essex, UK) resulting in a 1.3 μm axial resolution and a 2.2 μm lateral resolution.

In this study, OCT was mainly used as a real-time navigation tool to find the regions of interest (ROIs). At each ROI, a 562 μm by 562 μm by 1 mm OCT volume (averaged 5 times) was acquired. A flip mirror was then flipped down to switch to the NLOM modalities on the same field of view.

In order for OCT to be included in the present machine learning, the *en-face* slices corresponding to the planes imaged with the NLOM modalities were manually extracted from the OCT volumes and cropped so that the field of views matched with the two other modalities.

Although the final dataset was dominated by OCT with 59 out of 85 features, most of them were found to be non-significant for classification by the AutoML algorithm as only 10 were given a non-zero weight and OCT as a whole only accounted for 10.4% combined average weight of the algorithm. This indicates its predictive power appears to be small in comparison to both SHG and TPEF and it was therefore omitted from the main text.

However, this result should be interpreted carefully. Indeed, because OCT is an imaging technique sensitive to morphology, the sampling down of the images for features extraction may have had a more negative effect on it compared to the other modalities. Moreover, NLOM techniques produce 2D optical slices whereas OCT is intrinsically produces 3D volumes. In this study, only individual *en-face* slices taken from the OCT volumes were used for training the algorithm so that the images of all modalities could fit together on the same field of view but doing so fundamentally doesn't play to OCT's advantages and could explain the relative poor performances of the modality in this particular instance. In the future, an approach based on 3D radiomic features analysis of OCT volumes could be the key to unlock the predictive power of this modality, this however falls outside the scope of this work.

For the Correlation matrix and the Uniform Manifold Approximation and Projection (UMAP) views, see Figures 1 and 2.

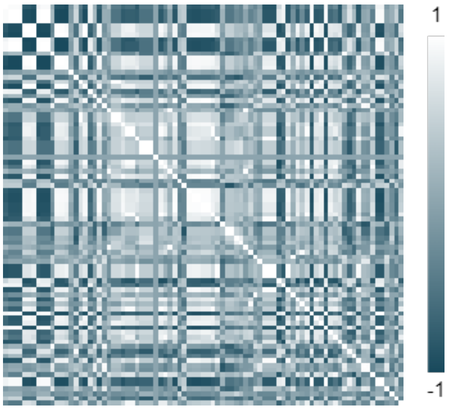

Figure 1. Correlation matrix view of the data (1). Correlation value 1 and -1 mean a 100% linear and inverse linear relationship between two features respectively. Feature pairs with near 0 correlation value are considered non-redundant.

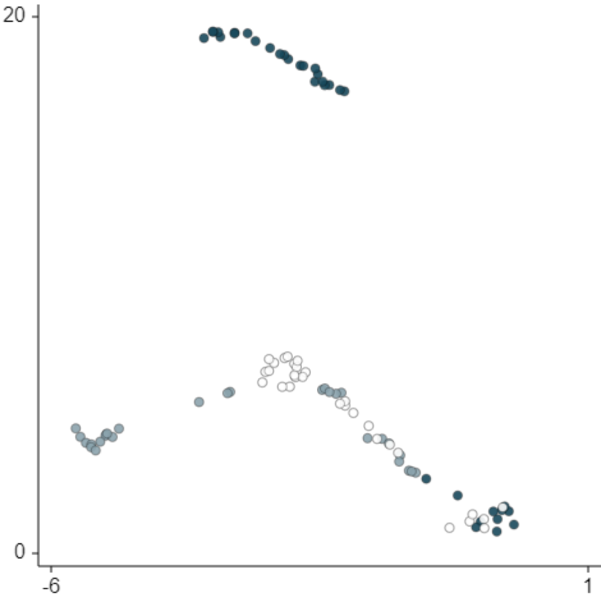

Figure 2. Uniform Manifold Approximation and Projection (UMAP) view of the data (2). Samples are colored by respective label outcomes.

Cross-Validation

Monte Carlo (MC) cross-validation scheme was applied with 80% training and 20% validation ratios across 100 folds (3). Each fold had unique training-validation configurations. MC split resulted in 74 samples per fold in the training set. The validation set of each fold contained 6 samples per reference label (18 overall). The validation samples were equally subsampled to ensure that none of the label outcomes are over or underrepresented during the cross-validation.

Preprocessing

The data underwent preprocessing steps in each fold before performing machine learning (ML) analysis. Preprocessing resulted in average 165 samples and 20 features across all MC folds. For the preprocessing steps and their parameters, see Table 2.

Table 2. Preprocessing step algorithms as well as their parameter values performed in all Monte Carlo folds before machine learning. RLB - Relabeling to binary labels; FN - Feature Normalization; SRR - Smart Redundancy Reduction; SSYN - Sample Synthetizer.

Preprocessing step	Algorithm	Parameter	Value	Reference
1	RLB	Label outcome to keep	0; 1; 2	–
2	FN	Normalization type	Mean-Deviation	(4)
3	SRR	Redundancy Threshold (Covariance)	0.85	(5)
4	SSYN	Oversampling ratio (majority subgroup)	1.55; 1.63	(6)
		Sampling technique	SMOTE	

Machine Learning Layer 1

Various machine learning algorithms were established in each fold to minimize the effect of algorithm bias (5). Each model was trained by randomly selecting 80% of the preprocessed training data per MC fold. For details of the ML algorithms, see Table 3.

Table 3. Machine learning (ML) algorithms in the first ML layer with their parameters and value ranges across Monte Carlo (MC) folds. Occurrence of each ML type is represented in percentages across MC folds. MGWC – Multi-Gaussian Weighted Classifier; RF – Random Forest Classifier; SVM – Support Vector Machine Classifier;

ML Algorithm	Parameter	Value Range	Occurrence	Reference
MGWC	Initial value multiplier	1 – 10	28.3%	(7)
	Maximum iterations	9500 – 40000		
	Negative weights allowed	false, true		
	Scale value multiplier	0.1 – 50		
	Tolerance	0.0001 – 0.001		

RF	Bag fraction	0.8 – 0.99	42.85%	(5)
	Bagging method	equalized, normal		
	Boosting	none, adaboost		
	Maximum tree depth	6 – 42		
	Minimum samples in leaves	3 – 6		
	Node feature selection method	none		
	Number of random features per node	6		
	Number of selected trees	101 – 201		
	Number of trees to build	301 – 1001		
	Tree quality metric	gain, gini		
	Tree selection method	0		
SVM	Learning rate	0.001 – 0.01	28.83%	(8)
	Maximum iterations	1000 – 5000		

Machine Learning Layer 2

Meta-training sets were created by evaluating the samples of the preprocessed training set in each MC fold by the trained models in ML layer 1. In order to create the meta-training set, the prediction results of each trained model in ML layer 1 were handled as feature values of the given training sample. The meta-training set was the input for training the second ML layer prediction models. These models were trained to identify patterns in the prediction of the first ML layer models to result in mixed super learners (9). For the parameters of the second layer ML algorithms see Table 4.

Table 4. Machine learning (ML) algorithms in the second ML layer with their parameters and value ranges across Monte Carlo (MC) folds. Occurrence of each ML type is represented in percentages across MC folds. MGWC – Multi-Gaussian Weighted Classifier;

ML Algorithm	Parameter	Value Range	Occurrence	Reference
MGWC	Initial value multiplier	10	100%	(7)
	Maximum iterations	9000 – 15000		
	Negative weights allowed	false, true		
	Scale value multiplier	1 – 5		
	Tolerance	0.0001		

Top-Layer Model

Combination of the prediction results of the second layer ML models was performed by weighted majority voting to provide the final prediction of the model scheme. Weighting of each ML Layer 2 model was calculated based on training performance. In addition, ML Layer 2 models having less training performance than the median of all ML layer 2 model training performances had weight 0 in the final vote.

RESULTS

Cross-Validation Performance

Model prediction performance was estimated via the MC cross-validation scheme utilizing confusion matrix analytics (10). True positive, true negative, false positive and false negative confusion matrix entries were calculated by evaluating the validation samples by the established model pipeline in each fold. Sensitivity, specificity, accuracy, positive predictive as well as negative predictive values were calculated across the MC fold validation results. For the average cross-validation performance of ML Layer 1 and 2 models see Table 5 and Table 6 respectively. For the cross-validation results of the final (top-layer) prediction models as well as for the summary of the evaluation, see Table 7 and Figure 3. For cross-validation area under the curve (AUC), see Figure 4.

Table 5. Average Monte Carlo (MC) cross-validation performance (%) of ML Layer 1 (ML-1) predictive models as determined by confusion matrix analytics across all MC folds. MGWC – Multi-Gaussian Weighted Classifier; RF – Random Forest Classifier; SVM – Support Vector Machine Classifier; SNS – Sensitivity; SPC – Specificity; PPV – Positive Predictive Value; NPV – Negative Predictive Value; ACC – Accuracy; OCC – Occurrence. Performance and occurrence values are in percentages.

	SNS	SPC	PPV	NPV	ACC	OCC
**MGWC**	98	75	90	93	90	28
**RF**	99	95	98	99	98	43
**SVM**	86	94	98	82	89	29

Table 6. Average Monte Carlo (MC) cross-validation performance (%) of ML Layer 2 (ML-2) predictive models as determined by confusion matrix analytics across all MC folds. MGWC – Multi-Gaussian Weighted Classifier; SNS – Sensitivity; SPC – Specificity; PPV – Positive Predictive Value; NPV – Negative Predictive Value; ACC – Accuracy; OCC – Occurrence. Performance and occurrence values are in percentages.

	SNS	SPC	PPV	NPV	ACC	OCC
**MGWC**	95	97	95	97	95	100

Table 7. Performance Monte Carlo (MC) cross-validation performance of the established model scheme throughout the performance of the top-layer prediction model. Performance values were determined by confusion matrix analytics across all MC folds. MGWC – Multi-Gaussian Weighted Classifier; SNS – Sensitivity; SPC – Specificity; PPV – Positive Predictive Value; NPV – Negative Predictive Value; ACC – Accuracy; AUC – Area Under the Receiver Operator Characteristics Curve. Performance values are in percentages. LQ – Lower quartile; UQ – Upper Quartile; Dev – Mean Deviation. 95% CI – 95% Confidence Interval.

	Min	LQ	Median	UQ	Max	Mean	Dev	95% CI
**SNS**	80	93.33	93.33	100	100	94.66	4.48	93.62 – 95.71
**SPC**	90	96.66	96.66	100	100	97.33	2.24	96.81 – 97.85
**PPV**	80	93.33	93.33	100	100	94.66	4.48	93.62 – 95.71
**NPV**	90	96.66	96.66	100	100	97.33	2.24	96.81 – 97.85

**ACC**	80	93.33	93.33	100	100	94.66	4.48	93.62 – 95.71
**AUC**	0	0	0	0	0	0	0	0 – 0

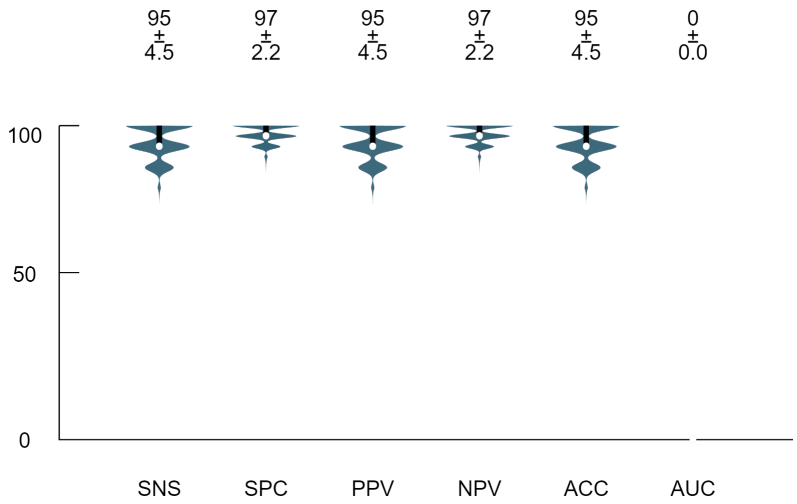

Figure 3. Box-plot Monte Carlo (MC) cross-validation performance of the established model scheme throughout the performance of the top-layer prediction model. Performance values were determined by confusion matrix analytics across all MC folds. SNS – Sensitivity; SPC – Specificity; PPV – Positive Predictive Value; NPV – Negative Predictive Value; ACC – Accuracy; AUC – Area Under the Receiver Operator Characteristics Curve. Performance values are in percentages.

Feature Importance

Feature ranking and selection was performed as part of the data preprocessing steps of each fold (see Sec. Preprocessing). The final feature importance was calculated as the mean of all feature rankings across the MC folds. Note that only the highest-ranking 30 features are shown in the table.

Table 8. Selected features and their ranks as calculated across the MC folds by Smart Redundancy Reduction (SRR - see Table 2) as well as their respective value distributions. Ranks represent the relative importance of selected features for model building. Features are ordered by ranks. Rank values are in percentages. LQ – Lower quartile; UQ – Upper Quartile; Dev – Mean Deviation.

Feature Name	Mean Ranking ±Dev	Min	LQ	Median	UQ	Max
		Feature Value Histogram				
TPEF::Histogram::ih.max	9.99% ±6.63%	0%	0%	13.97%	15.15%	19.14%
		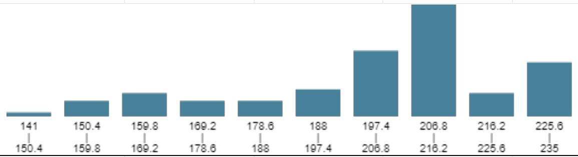				
		0%	1.19%	3.34%	15.32%	20.24%

TPEF::GLSZM::szm.lgze	6.76% ±6.44%	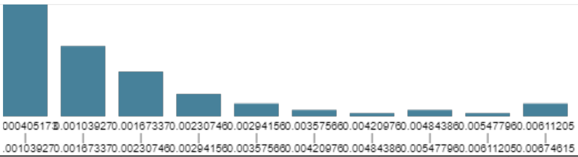				
		0%	0%	6.9%	11.88%	17.24%
TPEF::Histogram::ih.max.grad	6.66% ±4.48%	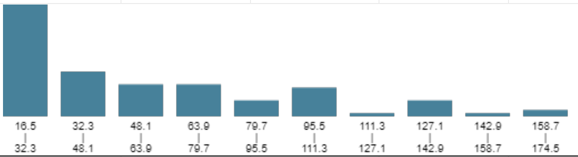				
		0%	0%	0.8%	18%	21.66%
SHG::NGLDM::ngl.dc.entr	6.58% ±8.27%	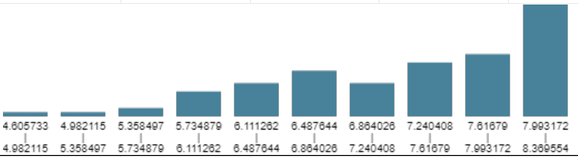				
		0%	0%	2.44%	13.44%	15.96%
SHG::GLRLM::rlm.lrhge	6.42% ±5.96%	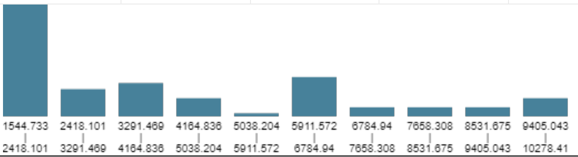				
		0%	0%	8.63%	9.53%	12%
TPEF::NGTDM::ntg.complexity	6.2% ±4.11%	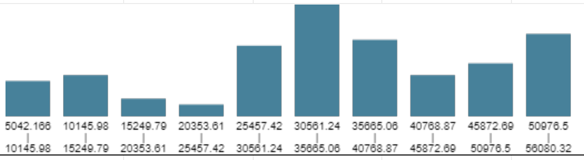				
		0%	0%	7%	10.37%	11.81%
SHG::Intensity::stat.min	5.85% ±3.98%	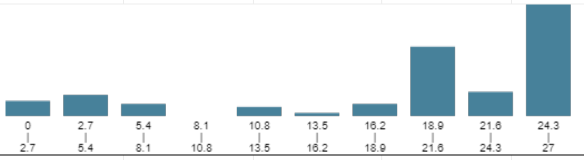				
		0%	0%	0%	13.1%	15.67%
SHG::Intensity::stat.mean	5.04% ±6.4%	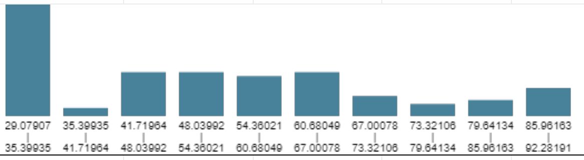				
		0%	0%	3.38%	7.31%	14.11%
SHG::IVHistogram::ivh.V90	4.73% ±3.79%	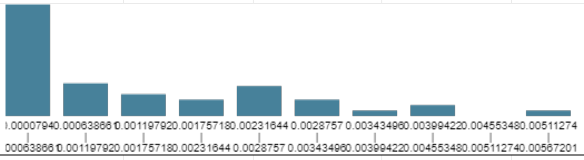				
		0%	0%	4.95%	8.8%	13.58%
TPEF::GLSZM::szm.zsnu	4.51% ±3.82%	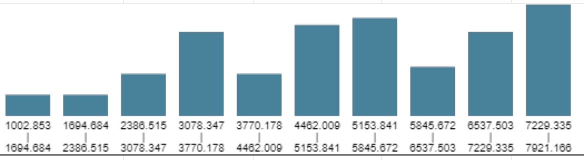				
		0%	0%	0%	12.47%	16.39%

SHG::Intensity::loc.peak.global	4.51% ±6.07%	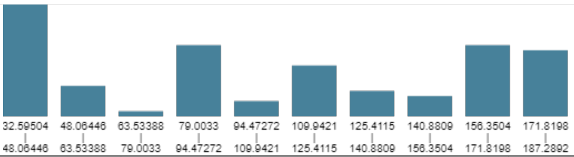
		0% 1.16% 5.36% 6.67% 9.18%
SHG::Histogram::ih.min.grad.gl	4.47% ±2.38%	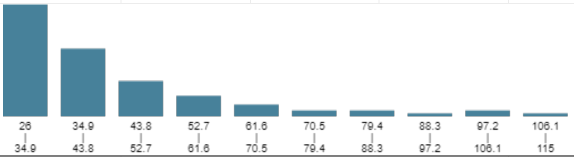
		0% 0% 0.68% 5.48% 17.73%
TPEF::GLCM::cm.joint.max	4.05% ±4.13%	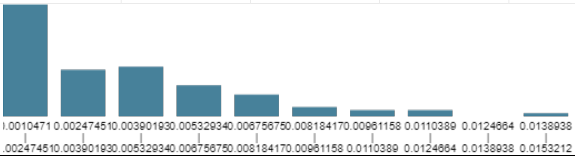
		0% 0% 1.37% 6.92% 13.19%
OCT::Histogram::ih.min.grad	3.17% ±3.18%	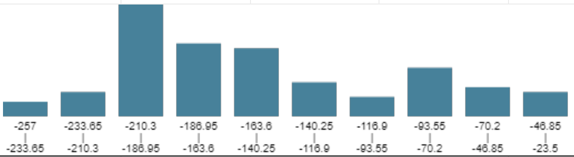
		0% 0% 3.21% 4.26% 8.51%
TPEF::Morphological::morph.av	2.72% ±1.95%	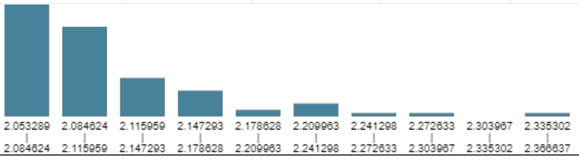
		0% 0% 1.48% 4.92% 7.57%
OCT::Intensity::stat.min	2.42% ±2.06%	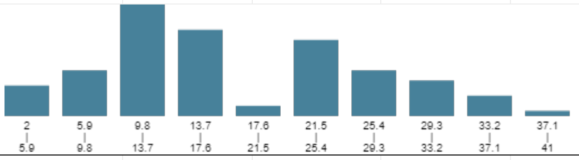
		0% 1.34% 2.11% 3.48% 5.54%
TPEF::GLCM::cm.inv.diff.mom.norm	2.39% ±1.1%	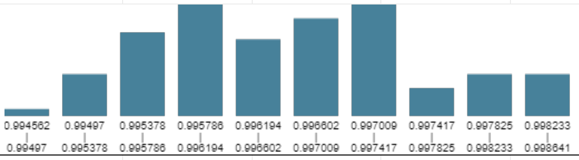
		0% 0% 0% 0% 11.34%
SHG::GLCM::cm.inv.diff.mom.norm	1.88% ±2.86%	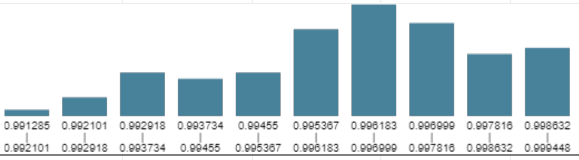
		0% 0% 0% 2.6% 6.08%
OCT::GLCM::cm.inv.diff.norm	1.28% ±1.36%	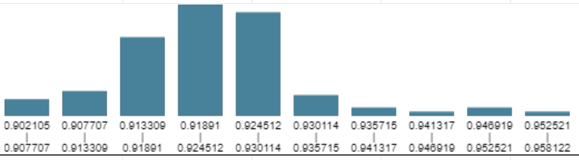
		0% 0% 0% 0% 12.85%

SHG::GLSZM::szm.zs.entr	1.27% ±2.23%	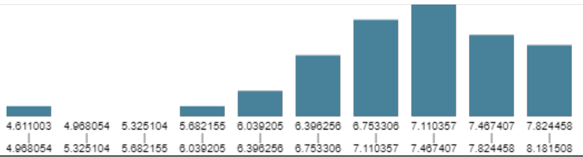				
		0%	0%	0%	3.23%	10.22%
SHG::Morphological::morph.com	1.23% ±1.63%	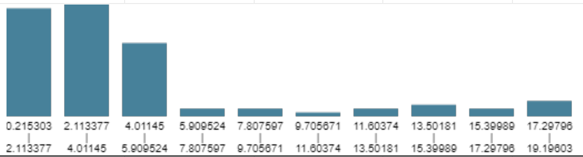				
		0%	0%	0.72%	1.18%	2.87%
OCT::Morphological::morph.com	0.68% ±0.57%	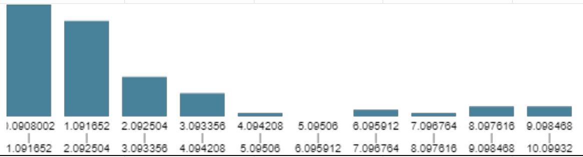				
		0%	0%	0.68%	1.06%	1.88%
SHG::GLCM::cm.corr	0.63% ±0.46%	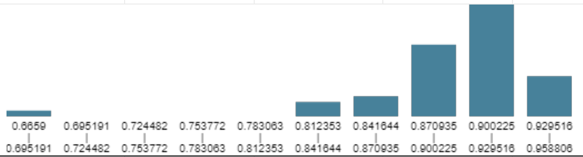				
		0%	0%	0%	1.32%	3.38%
OCT::Histogram::ih.max.grad.gl	0.61% ±0.74%	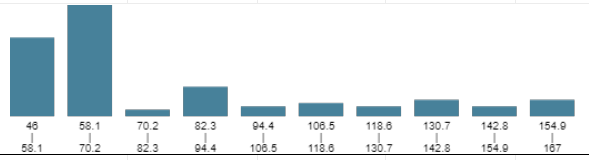				
		0%	0%	0.53%	0.88%	2.45%
OCT::GLSZM::szm.lzlge	0.53% ±0.48%	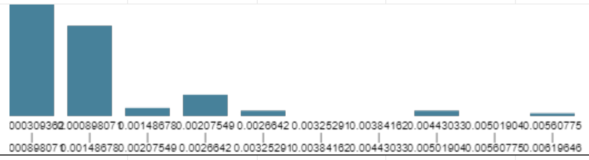				
		0%	0%	0%	0%	4.91%
OCT::GLSZM::szm.glnu	0.5% ±0.83%	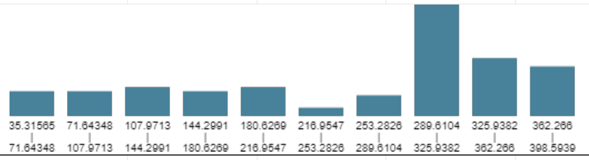				
		0%	0%	0.28%	0.94%	2.09%
TPEF::Histogram::ih.qcod	0.47% ±0.47%	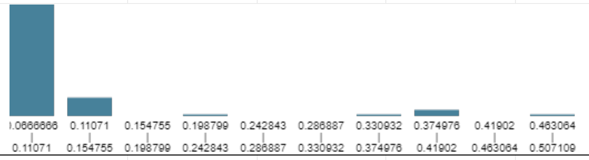				
		0%	0%	0%	0.24%	6.4%
OCT::Intensity::stat.median	0.42% ±0.63%	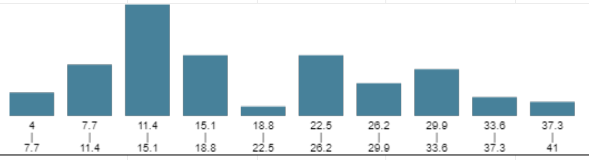				
		0%	0%	0.21%	0.77%	2.4%

<table>
<tr><td>OCT::IVHistogram::ivh.V90</td><td>0.42%
±0.43%</td><td>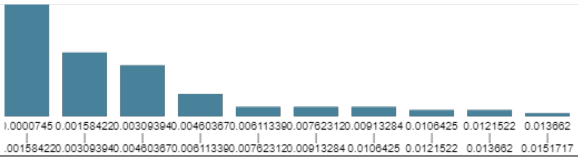
</td></tr>
<tr><td rowspan="2">OCT::NGLDM::ngl.hdhge</td><td rowspan="2">0.41%
±0.6%</td><td>0% | 0% | 0% | 0.73% | 2.44%</td></tr>
<tr><td>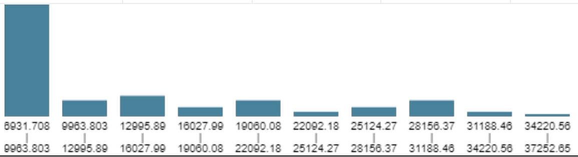
</td></tr>
</table>

References

1. Arita H, Kinoshita M, Kawaguchi A, Takahashi M, Narita Y, Terakawa Y, et al. Lesion location implemented magnetic resonance imaging radiomics for predicting IDH and TERT promoter mutations in grade II/III gliomas. Sci Rep [Internet]. 2018 Dec 6;8(1):11773. Available from: http://www.nature.com/articles/s41598-018-30273-4

2. McInnes, L, Healy, J, UMAP: Uniform Manifold Approximation and Projection for Dimension Reduction, ArXiv e-prints 1802.03426, 2018

3. Papp L, Spielvogel CP, Rausch I, Hacker M, Beyer T. Personalizing Medicine Through Hybrid Imaging and Medical Big Data Analysis. Front Phys [Internet]. 2018 Jun 7;6. Available from: https://www.frontiersin.org/article/10.3389/fphy.2018.00051/full

4. Han J, Pei J, Kamber M. Data Mining: Concepts and Techniques [Internet]. Elsevier Science; 2011. (The Morgan Kaufmann Series in Data Management Systems). Available from: https://books.google.at/books?id=pQws07tdpjoC

5. Papp L, Spielvogel CP, Grubmüller B, Grahovac M, Krajnc D, Ecsedi B, et al. Supervised machine learning enables non-invasive lesion characterization in primary prostate cancer with [68Ga]Ga-PSMA-11 PET/MRI. Eur J Nucl Med Mol Imaging [Internet]. 2020 Dec 19; Available from: http://link.springer.com/10.1007/s00259-020-05140-y

6. Amin A, Anwar S, Adnan A, Nawaz M, Howard N, Qadir J, et al. Comparing Oversampling Techniques to Handle the Class Imbalance Problem: A Customer Churn Prediction Case Study. IEEE Access. 2016;4(October):7940–57.

7. Papp L, Pötsch N, Grahovac M, Schmidbauer V, Woehrer A, Preusser M, et al. Glioma survival prediction with combined analysis of in vivo 11C-MET PET features, ex vivo features, and patient features by supervised machine learning. J Nucl Med. 2018;59(6):892–9.

8. Gao X, Chu C, Li Y, Lu P, Wang W, Liu W, et al. The method and efficacy of support vector machine classifiers based on texture features and multi-resolution histogram from18F-FDG PET-CT images for the evaluation of mediastinal lymph nodes in patients with lung cancer. Eur J Radiol [Internet]. 2015;84(2):312–7. Available from: http://dx.doi.org/10.1016/j.ejrad.2014.11.006

9. van der Laan MJ, Polley EC, Hubbard AE. Super Learner. Stat Appl Genet Mol Biol [Internet]. 2007 Jan 16;6(1). Available from: https://www.degruyter.com/view/j/sagmb.2007.6.issue-1/sagmb.2007.6.1.1309/sagmb.2007.6.1.1309.xml

10. Stehman S V. Selecting and interpreting measures of thematic classification accuracy. Remote Sens Environ [Internet]. 1997 Oct;62(1):77–89. Available from: https://linkinghub.elsevier.com/retrieve/pii/S0034425797000837

100-FOLD CONVENTIONAL-BASED AutoML

AUTOMATED MACHINE LEARNING ANALYSIS

Tabular data submission for Dedicaid[2] AutoML services was performed by user laszlo.papp@meduniwien.ac.at on 4/25/2024, 4:46:47 PM to build and cross-validate automated data preprocessing and mixed, stacked ensemble learning pipelines for predicting reference label label. For the details of the analysis see Table 1.

Table 1. Properties of the automated machine learning (AutoML) analysis of this study.

Data name	Gabriel-ConventionalF-Train.xlsx
Data size	92 samples, 16 features
Date of analysis	4/25/2024, 4:46:47 PM
Duration of analysis	6h 54m 6s
Submitted by	laszlo.papp@meduniwien.ac.at
Dedicaid AutoML version	0.1

METHODS

Data

The input dataset was composed of 92 samples and 18 features. The submitted dataset was composed of 16 features. The selected reference label for the cross-validation was label having subgroups of missing (32.6%) label outcomes.

For the Correlation matrix and the Uniform Manifold Approximation and Projection (UMAP) views, see Figures 1 and 2.

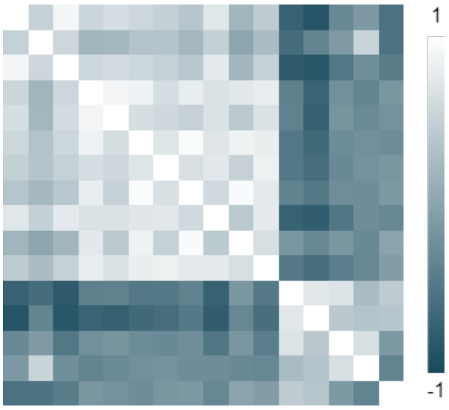

Figure 1. Correlation matrix view of the data (1). Correlation value 1 and -1 mean a 100% linear and inverse linear relationship between two features respectively. Feature pairs with near 0 correlation value are considered non-redundant.

[2]Dedicaid GmbH, a wholly owned subsidary of Telix Pharmaceuticals Limited

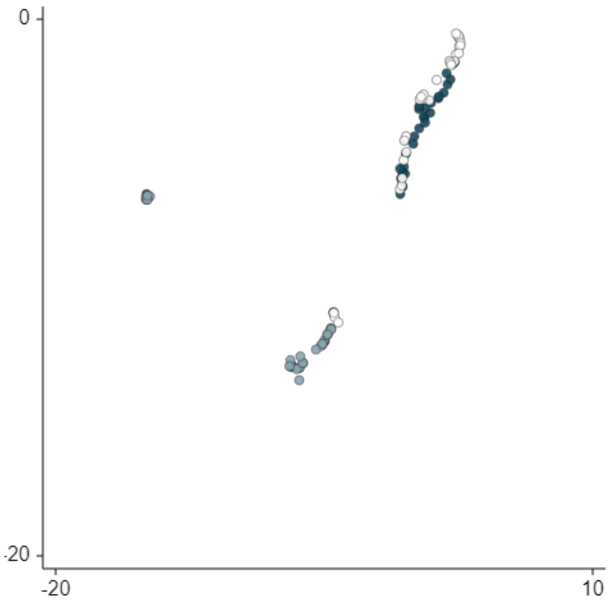

Figure 2. Uniform Manifold Approximation and Projection (UMAP) view of the data (2). Samples are colored by respective label outcomes.

Cross-Validation

Monte Carlo (MC) cross-validation scheme was applied with 80% training and 20% validation ratios across 100 folds (3). Each fold had unique training-validation configurations. MC split resulted in 74 samples per fold in the training set. The validation set of each fold contained 6 samples per reference label (18 overall). The validation samples were equally subsampled to ensure that none of the label outcomes are over or underrepresented during the cross-validation.

Preprocessing

The data underwent preprocessing steps in each fold before performing machine learning (ML) analysis. Preprocessing resulted in average 165 samples and 20 features across all MC folds. For the preprocessing steps and their parameters, see Table 2.

Table 2. Preprocessing step algorithms as well as their parameter values performed in all Monte Carlo folds before machine learning. RLB - Relabeling to binary labels; MDS - Missing Data Synthesis; FN - Feature Normalization; KE - Kernel-based Feature Engineering; SRR - Smart Redundancy Reduction; SSYN - Sample Synthetizer.

Preprocessing step	Algorithm	Parameter	Value	Reference
1	RLB	Label outcome to keep	0; 1; 2	–
2	MDS	–	–	(4)
3	FN	Normalization type	Mean-Deviation	(5)
4	KE	Kernels applied	Gaussian; Polynomial; Tanh	(6)
5	FN	Normalization type	Mean-Deviation	(5)

6	SRR	Redundancy Threshold (Covariance)	0.85	(7)
7	SSYN	Oversampling ratio (majority subgroup)	1.55; 1.63	(8)
		Sampling technique	SMOTE	

Machine Learning Layer 1

Various machine learning algorithms were established in each fold to minimize the effect of algorithm bias (7). Each model was trained by randomly selecting 80% of the preprocessed training data per MC fold. For details of the ML algorithms, see Table 3.

Table 3. Machine learning (ML) algorithms in the first ML layer with their parameters and value ranges across Monte Carlo (MC) folds. Occurrence of each ML type is represented in percentages across MC folds. MGWC – Multi-Gaussian Weighted Classifier; RF – Random Forest Classifier; SVM – Support Vector Machine Classifier;

ML Algorithm	Parameter	Value Range	Occurrence	Reference
MGWC	Initial value multiplier	1 – 10	32.98%	(9)
	Maximum iterations	9500 – 40000		
	Negative weights allowed	true, false		
	Scale value multiplier	0.1 – 50		
	Tolerance	0.0001 – 0.001		
RF	Bag fraction	0.8 – 0.99	36.62%	(7)
	Bagging method	normal, equalized		
	Boosting	adaboost, none		
	Maximum tree depth	5 – 8		
	Minimum samples in leaves	3 – 6		
	Node feature selection method	none		
	Number of random features per node	5		
	Number of selected trees	101 – 201		
	Number of trees to build	301 – 1001		
	Tree quality metric	gain, gini		
	Tree selection method	0		
SVM	Learning rate	0.001 – 0.01	30.38%	(10)
	Maximum iterations	1000 – 5000		

Machine Learning Layer 2

Meta-training sets were created by evaluating the samples of the preprocessed training set in each MC fold by the trained models in ML layer 1. In order to create the meta-training set, the prediction results of each trained model in ML layer 1 were handled as feature values of the given training sample. The meta-training set was the input for training the second ML layer prediction models. These models were trained to identify patterns in the prediction of the first

ML layer models to result in mixed super learners (11). For the parameters of the second layer ML algorithms see Table 4.

Table 4. Machine learning (ML) algorithms in the second ML layer with their parameters and value ranges across Monte Carlo (MC) folds. Occurrence of each ML type is represented in percentages across MC folds. MGWC – Multi-Gaussian Weighted Classifier;

ML Algorithm	Parameter	Value Range	Occurrence	Reference
MGWC	Initial value multiplier	10	100%	(9)
	Maximum iterations	9000 – 18000		
	Negative weights allowed	false, true		
	Scale value multiplier	1 – 5		
	Tolerance	0.0001		

Top-Layer Model

Combination of the prediction results of the second layer ML models was performed by weighted majority voting to provide the final prediction of the model scheme. Weighting of each ML Layer 2 model was calculated based on training performance. In addition, ML Layer 2 models having less training performance than the median of all ML layer 2 model training performances had weight 0 in the final vote.

RESULTS

Cross-Validation Performance

Model prediction performance was estimated via the MC cross-validation scheme utilizing confusion matrix analytics (12). True positive, true negative, false positive and false negative confusion matrix entries were calculated by evaluating the validation samples by the established model pipeline in each fold. Sensitivity, specificity, accuracy, positive predictive as well as negative predictive values were calculated across the MC fold validation results. For the average cross-validation performance of ML Layer 1 and 2 models see Table 5 and Table 6 respectively. For the cross-validation results of the final (top-layer) prediction models as well as for the summary of the evaluation, see Table 7 and Figure 3. For cross-validation area under the curve (AUC), see Figure 4.

Table 5. Average Monte Carlo (MC) cross-validation performance (%) of ML Layer 1 (ML-1) predictive models as determined by confusion matrix analytics across all MC folds. MGWC – Multi-Gaussian Weighted Classifier; RF – Random Forest Classifier; SVM – Support Vector Machine Classifier; SNS – Sensitivity; SPC – Specificity; PPV – Positive Predictive Value; NPV – Negative Predictive Value; ACC – Accuracy; OCC – Occurrence. Performance and occurrence values are in percentages.

	SNS	SPC	PPV	NPV	ACC	OCC
**MGWC**	96	81	91	93	91	33
**RF**	98	97	99	98	98	37

**SVM**	82	92	96	76	85	30

Table 6. Average Monte Carlo (MC) cross-validation performance (%) of ML Layer 2 (ML-2) predictive models as determined by confusion matrix analytics across all MC folds. MGWC – Multi-Gaussian Weighted Classifier; SNS – Sensitivity; SPC – Specificity; PPV – Positive Predictive Value; NPV – Negative Predictive Value; ACC – Accuracy; OCC – Occurrence. Performance and occurrence values are in percentages.

	SNS	SPC	PPV	NPV	ACC	OCC
**MGWC**	86	93	86	93	86	100

Table 7. Performance Monte Carlo (MC) cross-validation performance of the established model scheme throughout the performance of the top-layer prediction model. Performance values were determined by confusion matrix analytics across all MC folds. MGWC – Multi-Gaussian Weighted Classifier; SNS – Sensitivity; SPC – Specificity; PPV – Positive Predictive Value; NPV – Negative Predictive Value; ACC – Accuracy; AUC – Area Under the Receiver Operator Characteristics Curve. Performance values are in percentages. LQ – Lower quartile; UQ – Upper Quartile; Dev – Mean Deviation. 95% CI – 95% Confidence Interval.

	Min	LQ	Median	UQ	Max	Mean	Dev	95% CI
**SNS**	60	80	86.66	93.33	100	85.46	6.59	83.89 – 87.03
**SPC**	80	90	93.33	96.66	100	92.73	3.29	91.94 – 93.51
**PPV**	60	80	86.66	93.33	100	85.46	6.59	83.89 – 87.03
**NPV**	80	90	93.33	96.66	100	92.73	3.29	91.94 – 93.51
**ACC**	60	80	86.66	93.33	100	85.46	6.59	83.89 – 87.03
**AUC**	0	0	0	0	0	0	0	0 – 0

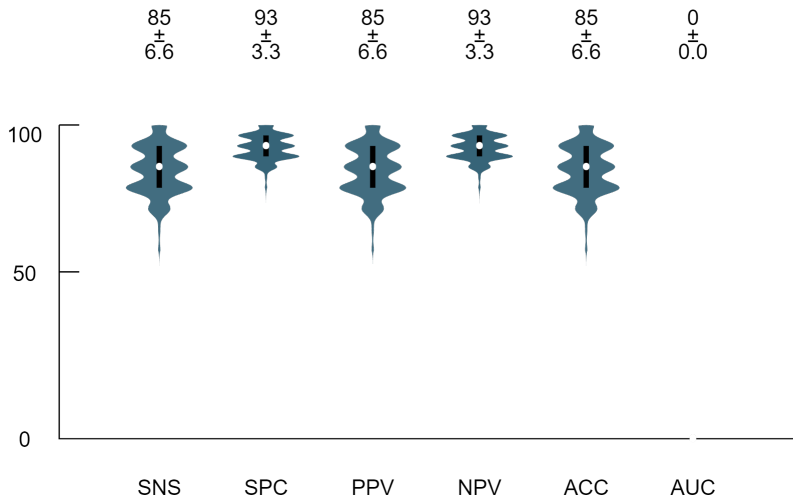

Figure 3. Box-plot Monte Carlo (MC) cross-validation performance of the established model scheme throughout the performance of the top-layer prediction model. Performance values were determined by confusion matrix analytics across all MC folds. SNS – Sensitivity; SPC – Specificity; PPV – Positive Predictive Value; NPV – Negative Predictive Value; ACC – Accuracy; AUC – Area Under the Receiver Operator Characteristics Curve. Performance values are in percentages.

Feature Importance

Feature ranking and selection was performed as part of the data preprocessing steps of each fold (see Sec. Preprocessing). The final feature importance was calculated as the mean of all feature rankings across the MC folds.

Table 8. Selected features and their ranks as calculated across the MC folds by Smart Redundancy Reduction (SRR - see Table 2) as well as their respective value distributions. Ranks represent the relative importance of selected features for model building. Features are ordered by ranks. Rank values are in percentages. LQ – Lower quartile; UQ – Upper Quartile; Dev – Mean Deviation.

Feature Name	Mean Ranking ±Dev	Min	LQ	Median	UQ	Max
		Feature Value Histogram				
Collagen amount, Intensity bas ed	13.23% ±5.8%	0%	7.9%	12.77%	17.99%	34.44%
		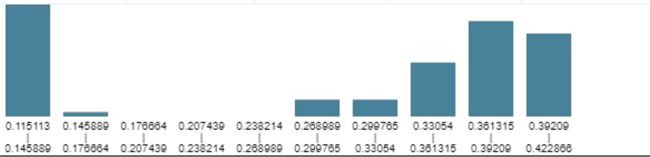				
Collagen amount, Segmentation based	11.55% ±5.67%	2.44%	4.52%	11.06%	17.04%	28.72%
		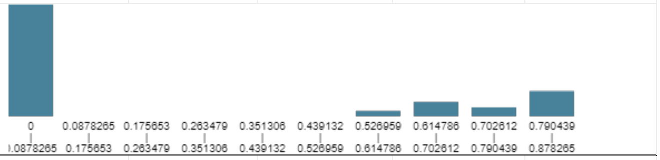				
SHG orientation, range	8.25% ±5.17%	0%	3.66%	9.32%	12.9%	22.47%
		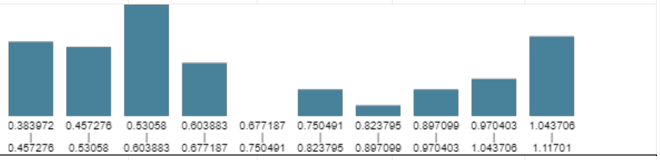				
Collagen amount, Number of fib res	8.06% ±3.61%	0%	5.31%	8.51%	11.38%	18.2%
		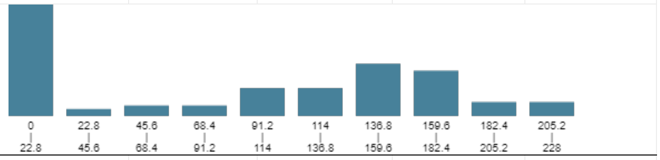				
Fibre morphology, avg widths	7.02% ±2.05%	1.98%	5.08%	6.39%	8.21%	16.74%
		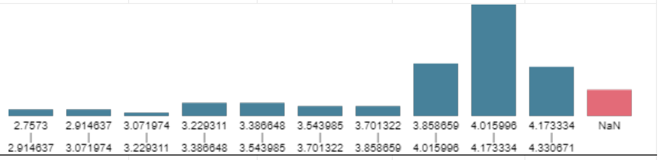				
TPEF orientation, range	6.53% ±3.37%	0%	1.79%	7.68%	9.38%	15.57%
		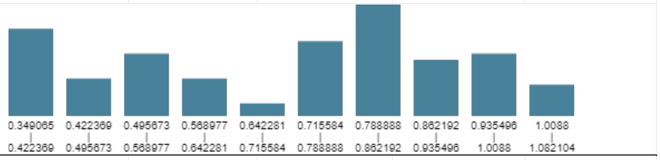				
		1.8%	4.38%	5.78%	7.75%	11.96%

Fibre orientation, MVL	6.11% ±1.85%	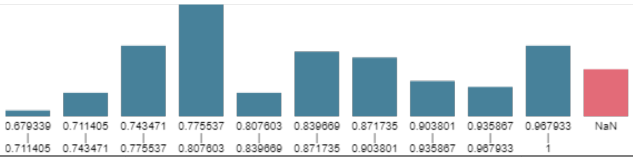				
		0%	3.52%	6%	7.96%	16.13%
SHG orientation, MVL	5.94% ±2.37%	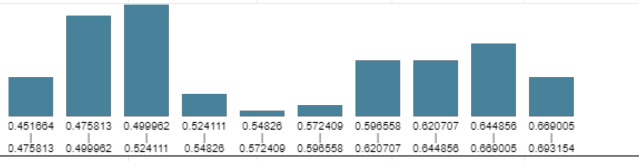				
		0.92%	4.61%	5.84%	7.19%	12.48%
SHG-TPEF MVA mismatch	5.7% ±1.56%	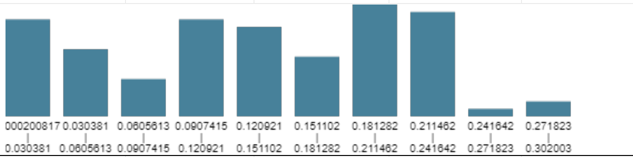				
		1.51%	4.44%	5.41%	6.62%	13.08%
Fibre morphology, std straight ness	5.67% ±1.47%	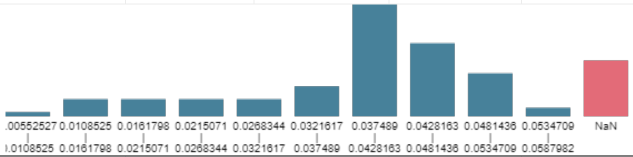				
		0%	3.07%	4.79%	7.83%	17.44%
Fibre morphology, std length	5.55% ±2.72%	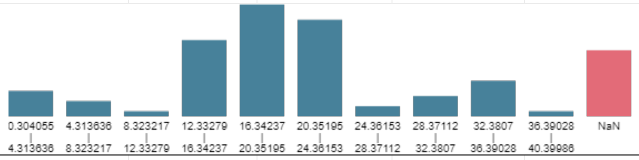				
		0%	2.78%	3.92%	6.05%	18.47%
Fibre morphology, avg length	4.51% ±2%	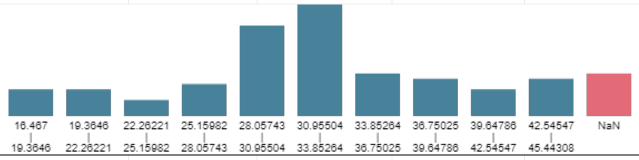				
		0.31%	2.43%	3.03%	4.06%	8.78%
Fibre morphology, std widths	3.3% ±1.04%	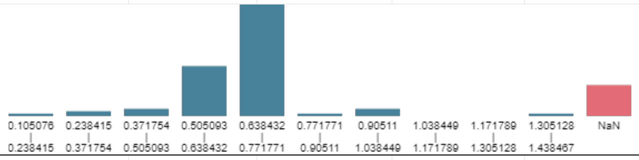				
		0%	2.27%	2.98%	3.86%	8.59%
Fibre morphology, avg straight ness	3.16% ±1.03%	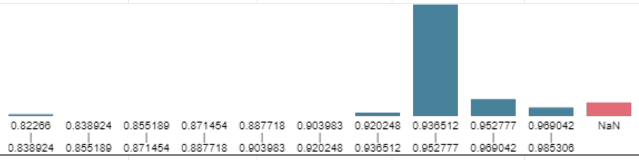				
		0.65%	2.05%	2.8%	3.68%	9.06%
Fibre orientation, range	2.99% ±1.01%	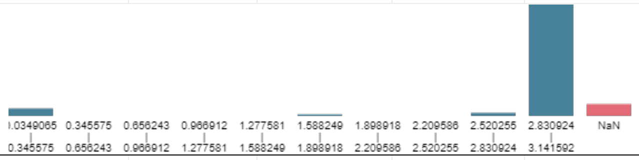				
		0%	1.46%	2.1%	3.1%	6.24%

TPEF orientation, MVL	2.35% ±0.95%	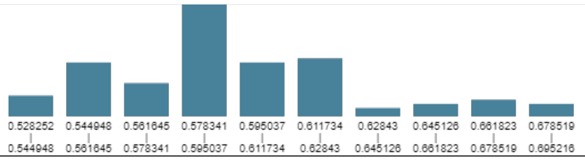

References

1. Arita H, Kinoshita M, Kawaguchi A, Takahashi M, Narita Y, Terakawa Y, et al. Lesion location implemented magnetic resonance imaging radiomics for predicting IDH and TERT promoter mutations in grade II/III gliomas. Sci Rep [Internet]. 2018 Dec 6;8(1):11773. Available from: http://www.nature.com/articles/s41598-018-30273-4

2. McInnes, L, Healy, J, UMAP: Uniform Manifold Approximation and Projection for Dimension Reduction, ArXiv e-prints 1802.03426, 2018

3. Papp L, Spielvogel CP, Rausch I, Hacker M, Beyer T. Personalizing Medicine Through Hybrid Imaging and Medical Big Data Analysis. Front Phys [Internet]. 2018 Jun 7;6. Available from: https://www.frontiersin.org/article/10.3389/fphy.2018.00051/full

4. Jakobsen JC, Gluud C, Wetterslev J, Winkel P. When and how should multiple imputation be used for handling missing data in randomised clinical trials – a practical guide with flowcharts. BMC Med Res Methodol [Internet]. 2017 Dec 6;17(1):162. Available from: https://bmcmedresmethodol.biomedcentral.com/articles/10.1186/s12874-017-0442-1

5. Han J, Pei J, Kamber M. Data Mining: Concepts and Techniques [Internet]. Elsevier Science; 2011. (The Morgan Kaufmann Series in Data Management Systems). Available from: https://books.google.at/books?id=pQws07tdpjoC

6. Souza CR. Kernel Functions for Machine Learning Applications [Internet]. 2020. Available from: http://crsouza.blogspot.com/2010/03/kernel-functions-for-machine-learning.html

7. Papp L, Spielvogel CP, Grubmüller B, Grahovac M, Krajnc D, Ecsedi B, et al. Supervised machine learning enables non-invasive lesion characterization in primary prostate cancer with [68Ga]Ga-PSMA-11 PET/MRI. Eur J Nucl Med Mol Imaging [Internet]. 2020 Dec 19; Available from: http://link.springer.com/10.1007/s00259-020-05140-y

8. Amin A, Anwar S, Adnan A, Nawaz M, Howard N, Qadir J, et al. Comparing Oversampling Techniques to Handle the Class Imbalance Problem: A Customer Churn Prediction Case Study. IEEE Access. 2016;4(October):7940–57.

9. Papp L, Pötsch N, Grahovac M, Schmidbauer V, Woehrer A, Preusser M, et al. Glioma survival prediction with combined analysis of in vivo 11C-MET PET features, ex vivo features, and patient features by supervised machine learning. J Nucl Med. 2018;59(6):892–9.

10. Gao X, Chu C, Li Y, Lu P, Wang W, Liu W, et al. The method and efficacy of support vector machine classifiers based on texture features and multi-resolution histogram from18F-FDG PET-CT images for the evaluation of mediastinal lymph nodes in patients with lung cancer. Eur J Radiol [Internet]. 2015;84(2):312–7. Available from: http://dx.doi.org/10.1016/j.ejrad.2014.11.006

11. van der Laan MJ, Polley EC, Hubbard AE. Super Learner. Stat Appl Genet Mol Biol [Internet]. 2007 Jan 16;6(1). Available from: https://www.degruyter.com/view/j/sagmb.2007.6.issue-1/sagmb.2007.6.1.1309/sagmb.2007.6.1.1309.xml

12. Stehman S V. Selecting and interpreting measures of thematic classification accuracy. Remote Sens Environ [Internet]. 1997 Oct;62(1):77–89. Available from: https://linkinghub.elsevier.com/retrieve/pii/S0034425797000837
